# Review of Research Advances in Gyroscopes’ Structural Forms and Processing Technologies Viewed from Performance Indices

**DOI:** 10.3390/s25196193

**Published:** 2025-10-06

**Authors:** Hang Luo, Hongbin Su, Qiwen Tang, Fazal ul Nisa, Liang He, Tao Zhang, Xiaoyu Liu, Zhen Liu

**Affiliations:** 1 Department of Measurement and Control Engineering, School of Mechanical Engineering, Sichuan University, Chengdu 610065, China; m15095542960@163.com (H.S.); tqw0328@163.com (Q.T.); 2022521411009@stu.scu.edu.cn (F.u.N.); hel20@scu.edu.cn (L.H.); nic6700@scu.edu.cn (T.Z.); liuxiaoyu@scu.edu.cn (X.L.); 2 School of Automation Engineering, University of Electronic Science and Technology of China, Chengdu 611731, China

**Keywords:** MEMS gyroscopes, Coriolis force, performance indices, dual mass, quadruple mass, hemisphere resonator gyroscope

## Abstract

As typical examples of rotational rate sensors, microelectromechanical system (MEMS) gyroscopes have been widely applied as inertial devices in various fields, including national defense, aerospace, healthcare, etc. This review systematically summarizes research advancements in MEMS gyroscope structural forms and processing technologies, which are evaluated through performance indices. The review encompasses several areas. First, it outlines the modelling principles and processes of gyroscopes on the basis of the Coriolis force and resonance, establishing a theoretical foundation for MEMS gyroscope development. Second, it introduces and analyzes the latest research advances in MEMS gyroscope structures and corresponding processing technologies. On the basis of published research advances, this review categorically discusses multidisciplinary technology properties, statistical results, the existence of errors, and compensation methods. Additionally, it identifies challenges in MEMS gyroscope technologies through classification analysis.

## 1. Introduction

With the continuous advancements in microelectronic design and fabrication, ultra-large-scale integrated circuits, and mechanical structure design, MEMS gyroscopes have become important inertial sensors for position, velocity, and acceleration measurements. They integrate technologies from multiple disciplines, including microelectronics, integrated circuits, mechanics, materials science, and control engineering, and have been widely applied in fields such as national defence, aerospace, aviation, marine engineering, mobile communications, geodetic surveying, geological exploration, automotive engineering, and medicine [1,2,3,4].

Despite the variety of structures and shapes, all MEMS gyroscopes are operated on the basis of the Coriolis force effect without exception. The development of micro/nanofabrication and architecture has made MEMS gyroscope research a focal area. Numerous researchers, universities [5,6,7,8,9,10,11], and companies [12,13,14,15,16] have conducted extensive studies, achieving significant progress in structural design and modelling [17,18,19,20,21,22,23,24,25,26,27], performance control and parameter optimization [28,29,30,31,32,33], precision enhancement [34,35,36,37], reliability analysis, and experimental validation [38,39,40,41].

On the basis of these research areas and achievements, the key focuses of MEMS gyroscope research can be categorized as follows [42].

(i) Structural design (coupled and uncoupled working modes, surface technology and processing, and encapsulation technologies), measurement, electronic circuit drive and control. This stage primarily involved verifying and improving the working principle of MEMS gyroscopes via closed-loop and open-loop detection methods. Processing limitations introduce nonrational errors in modal signals, affecting accuracy to some extent.

(ii) Correction of the control system through compensation and compound control strategies to reduce orthogonal error and feedback from detection forces to enhance dynamic and static performance.

(iii) Optimization of mode matching and the resonant frequency to improve precision, the signal-to-noise ratio, sensitivity and other performance metrics.

(iv) Reliability design, testing (including temperature stress and shock loading), modelling and estimation of the MEMS gyroscope system.

This review focuses on summarizing research advances in gyroscope structural forms and processing technologies from the perspective of performance indices. The remainder of this review is organized as follows: Section 2 introduces the principles of the Coriolis force effect, the working mechanism of the resonant gyroscope, and the evaluation indices. Section 3 discusses the stages of development and features of gyroscope research, analyzing typical structures and processing technologies along with their corresponding performances. Section 4 categorizes the impacts of design, fabrication, and other internal and external factors on performance. Section 5 summarizes and analyzes the key features and challenges of gyroscope technologies. Finally, the content and thread of the review are briefly presented in Section 6.

## 2. Basic Mechanics Principle and Indices of Gyroscope

### 2.1. Coriolis Force Effect and Coriolis Acceleration

**Figure 1:**
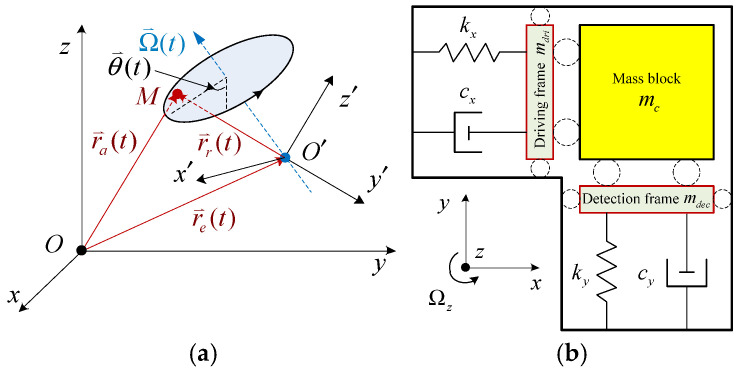
Fundamental principle and structure of a MEMS gyroscope: (**a**) schematic diagram for the derivation of Coriolis acceleration; (**b**) theoretical structure of a resonant MEMS gyroscope [43,44]. Printed with permission.

In a rotating coordinate system, a moving object undergoing radial or circular motion experiences an inertial force called the Coriolis force, which is orthogonal to the velocity of the rotating base. The Coriolis force arises from the relative motion and couples the rotating base and the relative moving object. Correspondingly, the Coriolis acceleration can be derived by studying the compound motion of a mass point (or rigid body). When analyzing compound motion, both fixed and moving coordinate systems must be considered simultaneously. Figure 1a shows the schematic diagram used to derive the Coriolis acceleration.

In Figure 1a, O-xyz represents a fixed coordinate system (also known as an inertial system), and O′-x′y′z′ denotes a moving coordinate system (also referred to as a noninertial frame) that rotates with an angular velocity Ω(t) (i.e., Ω(t) = θ′(t)). A mass point M undergoes relative motion within the moving coordinate system O′-x′y′z′. The absolute displacement vector r_a_(t) is defined with respect to the fixed coordinate system O-xyz, r_r_(t) is the relative displacement vector in O′-x′y′z′, and r_e_(t) represents the displacement vector of the moving coordinate system O′-x′y′z′. The absolute acceleration of mass point M can be derived via the following process.

The displacements r_a_(t), r_r_(t) and r_e_(t) satisfy the following relation:

(1) $$r_{a}(t)=r_{e}(t)+r_{r}(t).$$

By differentiating Equation (1) and considering the relative rotation of M with respect to the moving coordinate system O′-x′y′z′, a velocity relation can be obtained as follows.

(2) $${r_{a}}^{\prime }(t)={r_{e}}^{\prime }(t)+{r_{r}}^{\prime }(t)+\Omega (t)\times r_{r}(t),$$

where Ω(t) × r_r_(t) represents the tangential velocity due to the rotation of the coordinate system O′–x′y′z′. By performing a similar operation on Equation (2), an absolute acceleration relation can be derived as follows.

(3) $$r_{a}^{\prime \prime }(t)=r_{e}^{\prime \prime }(t)+r_{r}^{\prime \prime }(t)+\Omega (t)\times [\Omega (t)\times r_{r}(t)]+\Omega^{\prime }(t)\times {\vec{r}}_{r}(t)+2\Omega (t)\times r_{r}^{\prime }(t),$$

where r_e_″(t) denotes the radial acceleration of the moving frame O′-x′y′z′, r_r_″(t) denotes the relative radial acceleration with respect to the frame O′-x′y′z′, Ω(t) × [Ω(t) × r_r_(t)] represents the centripetal acceleration, Ω′(t) × r_r_(t) denotes the tangential acceleration, and 2Ω(t) × r_r_′(t) denotes Coriolis acceleration, which results from two components: one from changes in relative velocity caused by convected motion, and the other from the changes in convected velocity caused by relative motion. On the basis of this analysis, the absolute acceleration a(t) of the mass point M can be rewritten as follows:

(4) $$a(t)=a_{r}(t)+a_{e}(t)+a_{c}(t),$$

where a_r_(t) = r_r_″(t) denotes the relative acceleration, a_e_(t) = r_e_″(t) + Ω(t) × [Ω(t) × r_r_(t)] + Ω(t) × r_r_′(t) denotes the convected acceleration, and a_c_(t) = 2Ω(t) × r_r_′(t) denotes the Coriolis acceleration.

The Coriolis acceleration a_c_(t) indicates that when an object moves on a rotating base with angular velocity Ω(t), a new acceleration a_c_(t) with a modulus of 2|Ω(t) × r_r_′(t)| is generated, and its direction is perpendicular to the plane formed by the rotational angular velocity and the object’s relative velocity. Therefore, the rotating angular velocity of the moving base can be determined by detecting the Coriolis acceleration and relative velocity of the object that is on the moving base. This principle underlies all vibrator gyroscopes.

### 2.2. Principle of the Resonant Gyroscope

#### 2.2.1. Typical Structure of a Resonant MEMS Gyroscope

The typical theoretical structure of a resonant MEMS gyroscope is illustrated in Figure 1b [43,44]. In this configuration, the vibrating MEMS gyroscope can be modelled as two orthogonal spring-mass-damping systems sharing the same mass block m_c_, corresponding to the drive and detection structures.

When the system is operated, the driving force causes the mass block to vibrate along the direction of the *x*-axis (i.e., the driving axis). If the system rotates around the *z*-axis at a certain angular speed, the mass block experiences a Coriolis force along the *y*-axis (i.e., detection axis), thereby being induced to move along the detection axis.

#### 2.2.2. Principle of Resonance Detection

Considering the Coriolis forces in both the *x*-axis and *y*-axis directions, in conjunction with common models of Class II vibrating gyroscopes (which are the most representative among practical applications; other classes are beyond the scope of this work):

(5) $$\left\{\begin{array}{l} m_{x}x^{\prime \prime }+c_{x}x^{\prime }+k_{x}x=F_{dx}+F_{Coriolis\_x}\\ m_{y}y^{\prime \prime }+c_{y}y^{\prime }+k_{y}y=F_{Coriolis\_y}, \end{array}\right.$$

The Coriolis force term is as follows:

(6) $$\left\{\begin{array}{l} F_{Coriolis\_x}=-m_{c}\Omega_{z}y^{\prime }\\ F_{Coriolis\_y}=m_{c}\Omega_{z}x^{\prime }, \end{array}\right.$$

When the displacement is small or the dynamic behavior of the system can be simplified, the Coriolis force term can be neglected, yielding the following Equation:

(7) $$\left\{\begin{array}{l} m_{x}x^{\prime \prime }+c_{x}x^{\prime }+k_{x}x=F_{dx}\\ m_{y}y^{\prime \prime }+c_{y}y^{\prime }+k_{y}y=-2m_{c}\Omega_{z}x^{\prime }, \end{array}\right.$$

where m_x_ = m_c_ + m_dri_, m_y_ = m_c_ + m_dec_, and F_dx_ denotes the driving force along the *x*-axis.

Assuming that the driving force F_dx_ is a sine wave (i.e., F_dx_(t) = F_d·_sin(ω_d_t)) under steady-state conditions, the driving and detection modes exhibit simple harmonic vibrations, resulting in compound motion. When the driving angular frequency ω_d_ equals ω_x_, which is the resonant frequency of the driving mode determined by m_x_ and k_x_, the driving force achieves the maximum amplitude. Under this condition, the driving displacement and detection displacement can be expressed as follows:

(8) $$\left\{\begin{array}{l} x(t)=\frac{F_{d}Q_{x}}{m_{x}\omega_{d}^{2}}\cos\left(\omega_{d}t\right)=A_{x}\cos\left(\omega_{d}t\right)\\ y(t)=\frac{-2\Omega_{z}F_{d}Q_{x}}{m_{x}\omega_{d}\sqrt{\left(\omega_{y}^{2}-\omega_{d}^{2}\right)+\frac{m_{y}^{2}\omega_{d}^{2}}{Q_{y}^{2}}}}\sin\left(\omega_{d}t\right)=A_{y}\sin\left(\omega_{d}t\right), \end{array}\right.$$

where Q_x_ and Q_y_ are the quality factors of the driving and detection modes, respectively; ω_y_ represents the detection frequency, which is determined by m_y_ and k_y_; and A_x_ and A_y_ are the corresponding amplitudes. According to Equation (8), the mechanical sensitivity of the resonant MEMS gyroscope can be defined as follows:

(9) $$S=\frac{A_{y}}{\Omega_{z}}\approx \frac{-F_{d}Q_{x}}{m_{x}\omega_{d}^{2}\left(\omega_{y}-\omega_{d}\right)}=\frac{-A_{x}}{\Delta \omega }.$$

where ∆ω represents the value of the angular frequency between the driving and detection angular frequencies. According to Equations (8) and (9), the following conclusions can be obtained:

- The detection amplitude is directly proportional to the rotation angular velocity of the base (system).
- The mechanical sensitivity of the system is proportional to the amplitude of the driving mode and inversely proportional to the difference in frequency between the driving frequency ω_d_ and the detection frequency ω_y_.
- Mechanical sensitivity can be enhanced by increasing the driving quality factor, deducing the driving mass, and decreasing the difference frequency.

Two types of mechanical structures are commonly adopted on the basis of measurement requirements: degeneracy and nondegeneracy working modes [10]. In degenerate mode, the difference in frequency between the driving and detection frequencies is zero, whereas in nondegenerate mode, this difference is nonzero.

The basic working principles and performance influencing factors outlined above suggest that the exploration direction of MEMS gyroscopes should be guided by a series of evaluation indices. Standardized performance metrics serve as evaluation criteria, providing users with references for comparison, evaluation and application.

### 2.3. Performance Indices of the Gyroscope

The key performance indices of the gyroscope are described below [45]:

#### 2.3.1. Scale Factor

The scale factor is defined as the ratio of the output voltage amplitude to the angular velocity of the input rotation. It represents the slope of a line obtained through least squares fitting of input-output data across the entire range of input angular rates. The residual error from this fitting determines the credibility of the fitted data and reflects the degree of deviation from the actual input-output data of the gyro. Common units for the scale factor include mV/(°/s) and LSB/(°/s).

#### 2.3.2. Threshold Value/Resolution

The threshold value indicates the minimum input angular rate to which the gyroscope can respond, whereas resolution refers to the smallest detectable change in the input angular rate at a specified rate. Both indices reflect the sensitivity of the gyroscope. Resolution is typically given in terms of bandwidth.

#### 2.3.3. Measuring Range

The measurement range represents the span of the input angular rates from the positive maximum value to the negative maximum value. The dynamic range, defined as the ratio of the maximum measurable range to the threshold value, indicates the gyroscope’s ability to detect varying rates. A larger dynamic range signifies better sensitivity.

#### 2.3.4. Zero-Bias Stability

Zero-bias stability (or Zero Drift, ZRO) refers to the steady-state output of the gyroscope when no input is present. This output fluctuates around a mean value over time, representing a stationary random process. The root mean square (RMS) error of this process defines zero-bias stability, which is typically measured in °/h (or °/s).

#### 2.3.5. Angle Random Walk (ARW)

ARW evaluates the white noise level of angular motion by quantifying the accumulated error coefficient in the gyroscope’s output due to white noise. ARW indirectly indicates the minimum detectable angular rate limited by the effects of particle noise, reflecting the overall quality of the gyroscope. The common units for ARW are °/√h or °/s•√Hz.

#### 2.3.6. Band Width (BW)

BW refers to the frequency range within which the gyroscope can accurately measure the input angular rate. A wider bandwidth enhances the gyroscope’s dynamic response capability.

In addition to these primary indices, other indices, such as the quality factor (Q-factor), structural height, and impact resistance, also contribute to evaluating the design quality of MEMS gyroscopes. On the basis of these performance indices, MEMS gyroscopes can be classified into rate-grade, tactic-grade, and inertial-grade categories for different applications [45].

On the basis of the above principles and performance indices, research in the field of MEMS gyroscopes has focused on structural design, parameter optimization, operational control, reliability design, analysis and evaluation, forming a broad research field.

### 2.4. Quantitative Analysis of the Impact of MEMS Gyroscope Structures, Closed-Loop Control, and Phase Alignment Accuracy

We conducted a detailed quantitative analysis of the impact of different MEMS gyroscope structures, closed-loop control, and phase alignment accuracy, which are shown as in Table 1, Table 2, and Table 3 respectively. The specifics are as follows.

As shown in Table 1, the structural form of MEMS gyroscopes significantly influences their performance characteristics. Vibratory structures [8] generally exhibit higher sensitivity (e.g., 27.6 mV/°/s) but maintain a relatively low noise density, making them suitable for high-precision applications that tolerate some noise. Tuning fork structures [46] provides a higher sensitivity (23 mV/°/s) with low noise and nonlinearity, offering enhanced stability. The impact of phase alignment precision and structural selection on performance metrics varies depending on the application, with certain designs being more effective in specific performance domains.

Closed-loop control strategies are critical in enhancing MEMS gyroscope performance. Viewed from Table 2, PID control [51] significantly reduces overshoot (96%) and shortens the settling time, making it suitable for applications with temperature disturbances. LQR control [47] improves system stability, reduces noise, and allows for bandwidth optimization. PLL-based control [50] effectively minimizes ZRO drift and enhances system stability and precision. The choice of control strategy should be based on the specific performance requirements, as each method offers distinct advantages.

The alignment accuracy of the phase plays a significant role in the performance of MEMS gyroscopes. According to the information shown in Table 3, phase errors [50] as large as 19.419 ± 0.004° can lead to a substantial reduction in ZRO (755%) and improve system stability. PLL-based phase correction [47] can significantly reduce ZRO drift, thus enhancing noise performance. However, phase errors exceeding a critical threshold may severely degrade the precision and stability of the system. Therefore, maintaining accurate phase alignment is essential for high-performance MEMS gyroscopes.

## 3. Research Advances in the Field of Gyroscopes’ Structural Form and Processing Technologies

In this section, the evolution and fundamental characteristics of gyroscope research are first introduced. Subsequently, an in-depth analysis and review of the research advancements in MEMS gyroscopes are provided, focusing on their structural forms, processing technologies, and corresponding performance metrics.

### 3.1. Typical Development of Gyroscope

#### 3.1.1. Division of Structural Development Stages

Since the invention of the first gyroscope—a universal joint gyroscope made by Foucault in 1852—the design and functionality of gyroscopes have continuously evolved, following different technological routes. The development can be outlined as follows:

(i) Rotating mechanical gyroscopes (1850s–20th century)

Traditional gyroscopes were based on high-speed rotating rotors. They were widely used in navigation, but suffered from problems such as mechanical wear, mass imbalance, and large size.

(ii) Optical gyroscopes (1970s–present)

With the emergence of ring laser gyroscopes (RLGs) and fibre-optic gyroscopes (FOGs), optical gyros became important for aerospace and navigation systems, providing high precision without moving parts.

(iii) Vibrating structure gyroscopes (1960s–present)

Quartz tuning-fork gyroscopes and hemispherical resonator gyroscopes (HRGs) detect Coriolis forces in vibrating structures. They offer high reliability and long lifetimes, although some designs require costly precision fabrication.

(iv) MEMS gyroscopes (1980s–present)

MEMS gyroscopes, which are the main focus of this work, benefit from advances in microelectronics and micromachining. They are compact, low-cost, and suitable for mass production, enabling applications in consumer electronics, automotive systems, and portable devices.

(v) New concepts (2000s–present)

Recent developments include atomic gyroscopes and surface acoustic wave (SAW) gyroscopes, which exploit quantum or acoustic effects. These technologies aim at achieving extreme sensitivity and long-term stability.

#### 3.1.2. Several Impacts Caused by Structural Evolution

(i) Relationship between structural evolution and performance

In the historical development of gyroscopes, changes in structural configuration have directly influenced performance improvements. From early mechanical gyroscopes to modern MEMS gyroscopes, structural design has undergone significant evolution. We have summarized the key milestones of these evolutions and, through the introduction of specific technologies, explained how they have driven the optimization of gyroscope performance. For example, the MEMS gyroscope sensitive element based on SOI technology uses an open-loop structure and integrates dedicated circuits, which not only enhances dynamic response characteristics but also improves accuracy, leading to significant advancements in gyroscope performance [54].

(ii) Biomimetic design and structural innovation

Furthermore, the introduction of biomimetic structures has provided a new direction for multi-axis angular velocity measurement in MEMS gyroscopes. By mimicking the design of insect balancers, a three-axis biomimetic gyroscope successfully measures three-axis angular velocity. This structural innovation has expanded the application range of gyroscopes and offered new perspectives on structural evolution [55]. We believe that advances in biomimetic design not only provide new solutions for structural evolution but also further increase the capability of gyroscopes in complex environments.

(iii) Impact-Resistant and Thermal-Stress-Optimized structural improvements:

To improve the impact resistance of MEMS gyroscopes, the introduction of a dual-mass design has significantly enhanced their shock resistance. By adjusting the in-phase frequency and incorporating a dual-stage elastic stopper mechanism, the MEMS gyroscope’s shock resistance has been improved, ensuring its high reliability and stability [56]. Additionally, research based on cantilever plate optimization design shows that the impact of thermal stress on MEMS gyroscope performance has been effectively reduced, further enhancing stability under different temperature conditions [57].

The structural evolution of gyroscopes is reflected not only in innovations in sensitive elements but also in aspects such as biomimetic design, improved impact resistance, and thermal stress optimization. These research advancements provide a valuable theoretical foundation for the future design and application of MEMS gyroscopes and demonstrate the close relationship between structure and performance.

### 3.2. Performance of Different Structure Forms and Machining Processing in MEMS Gyroscopes

Advancements in material processing technology have enabled the use of various materials, such as crystalline silicon [32], poly-silicon [7], fused quartz [33], silicon dioxide [8], and diamond [6], for designing MEMS resonator gyroscopes, making them active research topics. MEMS resonator gyroscopes can be classified into three-dimensional and surface structures, including mass block gyroscopes [31], ring gyroscopes, hemispherical gyroscopes [29], and cylindrical gyroscopes [5].

We investigate, analyze, and summarize the typical structure and processing technologies of gyroscopes, focusing on mass block, ring, and hemispherical types. Specifically, mass block gyroscopes exhibit linear vibration, whereas ring and hemispherical gyroscopes utilize standing wave vibration.

#### 3.2.1. Single-Mass Block Configuration

In the early stages of development, the sensitive mass block of a MEMS gyroscope was typically a single-mass block, with capacitance detection serving as the primary output method. This approach has inherent limitations such as low sensitivity, high complexity in structural design and fabrication, and a relatively small signal-to-noise ratio due to the micro-sizing effect, complex closed-loop circuits, and electromechanical coupling. The exploration of new structures and detection methods for MEMS gyroscopes has been a key driver for the diversification of structural and processing techniques.

**Figure 2:**
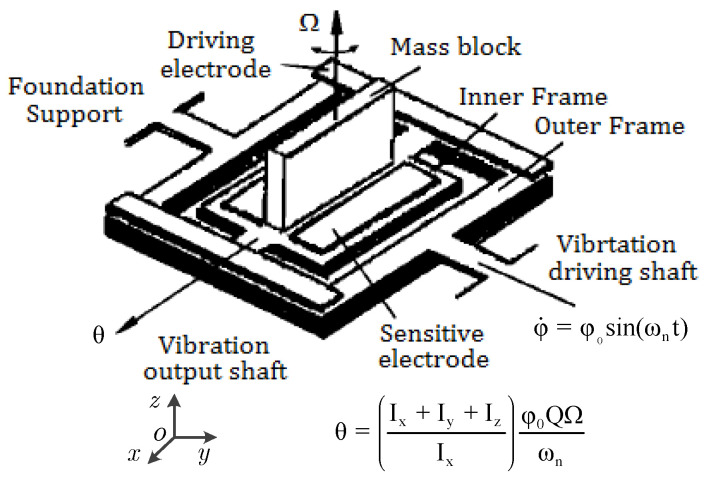
Double-frame gyroscope designed by the Draper Lab [58]. Printed with permission.

As illustrated in Figure 2, the first MEMS gyroscope featured a sensitive mass block supported by two mutually perpendicular frames connected via torsional axes. This design achieved an angular resolution of 4°/s at a BW of 1 Hz [58].

According to the defined mechanical constraints shown in Figure 2, the input angular rate Ω is determined by the output angle θ; the moments of inertia around the *x*-, *y*- and *z*-axes; the drive angle φ_0;_ and the mechanical Q.

A comb-drive gyroscope fabricated via surface silicon processing and ion etching technologies, with adjustable structural parameters to match resonant frequencies, can achieve an equivalent noise angular velocity of 2°/s under 1 Pa atmosphere pressure [59].

A single-mass-block MEMS gyroscope with independent external (driving) and internal (detection) modes was designed to isolate the coupling between detection and driving modes, reducing the orthogonal error. This design achieved a zero-bias stability of 0.07°/s at a BW of 10 Hz [60]. On the basis of the Differentially Accelerated Vertical Electrostatic Drive (DAVED) principle and the use of double-ended tuning fork (DETF) technology on a SOI, two types of decoupled gyroscopes (LL-structure, RR-structure, LR-structure, and others) with a capacitance detection method were realized. Both achieved a scale factor of 10 mV/(°/s) and a resolution of 0.005°/s at a bandwidth of 50 Hz. The LL structure enabled a range of ±100°/s, an RMS noise of 0.025°/s, and a nonlinearity of less than 0.1%, whereas the RR structure enabled a range of ±200°/s, an RMS noise of 0.05°/s, and a nonlinearity of less than 0.05% [61].

A symmetric gyroscope produced via complementary metal–oxide–semiconductor (CMOS), surface and bulk silicon micromachining, and the Lithographie, Galvanoformung, Abformung (LIGA) process achieved a quality factor of 10,400, a resolution ratio of 1.6°/s at 10 mTorr, an ARW of 438°/√h, and a resolution ratio of 96°/s under vacuum at a BW of 50 Hz [62]. An improved gyroscope with a two-stage elastic folded beam structure fabricated via a CMOS-compatible nickel electroplating process achieved a noise equivalent rate of 6°/√h, a short-term bias stability of better than 0.1°/s, a scale factor of 17.7 mV/(°/s) within a measuring range of ±100°/s, and a nonlinearity of less than 0.12% by matching the resonance frequencies of the driving and detection modes [63]. A single-mass MEMS gyroscope fabricated via silicon-glass bonding and a deep reactive ion etching process achieved a measurement range of ±300°/s, a scale factor of 20 mV/(°/s), and a nonlinearity of 0.56% [64].

Under high-frequency vibration conditions, single-mass-block MEMS gyroscopes present significant issues such as noise interference, mechanical loss, and sensitivity to external acceleration, leading to measurement errors. For example, shock or random vibrations can be mistaken for normal excitation, complicating the distinction and mitigating environmental interference, thus reducing the detection accuracy and limiting practical applications. To address these challenges, various compensation and correction technologies have been employed, including push–pull driving, differential detection, closed-loop control, phase-locked loop control and automatic gain control [65,66], orthogonal correction and PID correction [67,68], to increase the driving force, suppress common mode interference, and match frequency modes [28].

By designing novel mass block configurations and employing specific driving and sensing methods under advanced fabrication and processing technologies, some improved single-mass-block gyroscopes have demonstrated superior performance. A gyroscope with a large mass and capacitance, fabricated via silicon–glass wafer bonding and a deep trench etching process, achieved an ARW of 0.05°/(h•√Hz) [69]. On the basis of this design, a three-mass block configuration (with two small mass blocks connected to one-dimensional springs used as driving and sensing masses, and the large mass is taken as the proof mass) achieves a scale factor of 22 mV/(°/s), a nonlinearity of 2.19%, and an ARW of 1.2°/√h [70]. Additionally, by using a dicing-free fabrication process, a similar gyroscope, designed with a triple-mass scheme to achieve decoupled mode and a matched vibration structure, was reported to achieve a resolution (i.e., ARW) of 2.52°/(h•√Hz) [71].

A bulk silicon gyroscope featuring a novel composite cantilever beam–mass structure fabricated via novel masked-maskless etching technology on a horizontal beam was described to achieve a scale factor of 0.2 μV/(°/s) [72]. An improved gyroscope with a cantilever beam–mass structure enabled a sensitivity of 11.45 μV/(°/s) for angular rate detection via amplitude measurement and a phase change of 0.152°/s within a measurement range of ±120°/s [73].

#### 3.2.2. Dual-Mass-Block Configuration

The structure of a dual-mass MEMS gyroscope consists of two identical single-mass units in full symmetry. Unlike single-mass configurations, this design includes coupling beams connecting the two mass blocks in addition to driving and detection beams of each mass block. The coupling beams significantly influence the vibration characteristics of the microgyroscope structure. In the design of dual-mass-block microgyroscopes, coupling mechanisms are often employed to connect two mass blocks to ensure identical natural frequencies between the two masses. Through fully decoupled design, mutual interference between the driving and detection modes can be effectively minimized, allowing independent analysis of the driving mode or detection mode as a dynamic system with two degrees of freedom.

Although the dynamics principle of a dual-mass MEMS gyroscope is based on that of a single-mass MEMS gyroscope, the structure itself of the former is less susceptible to environmental factors than that of the latter. Like single-mass-block structures, differential detection is commonly adopted in dual-mass MEMS gyroscopes to suppress common mode interference signals and mitigate the effects of temperature and mechanical environments. In this type of structure, a tuning fork commonly serves as both a deriving structure and a detection structure, achieving high vibration Q values and effective vibration decoupling from the shell or frame [74].

**Figure 3:**
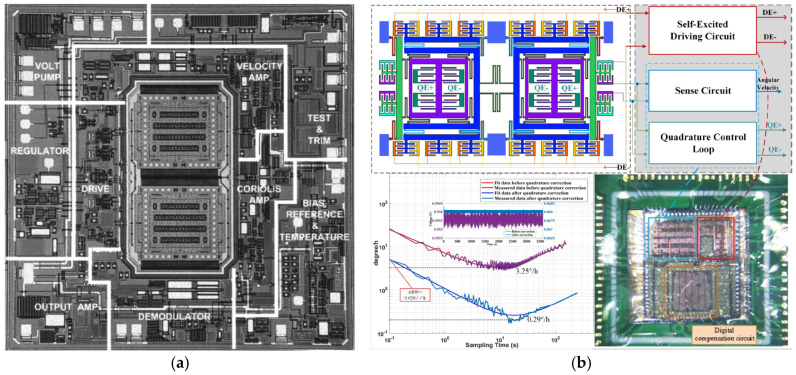
Typical dual-mass-block structures: (**a**) The structure diagram of the dual-mass MEMS gyroscope investigated [75]; (**b**) principle, test and photograph of monolithic integrated interface ASIC with quadrature error correction for a MEMS dual-mass vibration gyroscope [76]. Printed with permission.

As shown in Figure 3a, a dual-mass surface micromachined gyroscope, fabricated via a 3-μm BiCMOS process on a polysilicon structure, was reported to enable a scale factor of 12.5 mV/(°/s), an ARW of 3°/h, a full-scale range of ±150°/s and a BW of 100 Hz [75]. This was the first commercial gyroscope to gain significant market share at a lower price because of its excellent shock immunity (surviving shocks over 33,000 gee) and operational performance during 1000 gee shocks, resulting in an integrated angle error of less than 0.03°.

According to Honeywell reports, on the basis of a series of dual-mass tuning fork MEMS gyroscope technologies, the HG1900 series navigation systems achieved a zero repeatability of 12°/h, a zero-bias stability of 1.6°/h, and an ARW of 0.047°/√h [77]. On the basis of the impact of vibration frequency differences between two sensitive mass directions, a design concept of “Match-Mode” was proposed to increase instrument accuracy [78]. Further application of “Match-Mode” in a closed-loop servo circuit resulted in a zero-bias stability of 0.2°/h and an ARW of 0.18°/(h•√Hz) in the new instrument [79]. Subsequent research, which utilized residual quadrature error on a tuning fork gyroscope, achieved mode matching at an approximately 0 Hz split between the high-Q drive and sense mode frequencies by adopting a control algorithm interfaced with a CMOS integrated circuit (IC), resulting in a standard 0.6-μm process with a die area of 2.25 mm^2^, consuming 6 mW of power. The mode-matched gyroscope achieved a bias drift of 0.18°/h, a sensor Q of 36,000, a maximum scale factor of 88 mV/(°/s), and a varied BW between 1 Hz and 10 Hz [32].

By integrating amplitude-amplifying techniques with noise mitigation technologies, which include precision electrostatic frequency tuning, increased sensing capacitance and a low-noise front-end electronics design with balanced and differential sensor channels, an amplitude-amplifying dual-mass gyroscope was developed. This device features an internal mass block for drive functionality and an external mass block for sensing, connected via a concentric ring cantilever beam. The design achieved a zero-bias stability of 0.09°/h and an ARW of 0.096°/√h, significantly enhancing the signal-to-noise ratio of the device [80].

A fully decoupled *z*-axis MEMS gyroscope, fabricated via a two-mask process on a SOI substrate with a device layer of 30 μm, was demonstrated to achieve a bias instability of 9.6°/h, an ARW of 0.45°/√h, and a full-scale range of ±500°/s at room temperature [81]. To address the issue of in-phase mode vibrations causing electrostatic nonlinearities due to mechanical end collisions, a control method was developed using electronic circuits to correct the electrostatic balance between the two masses of a dual-mass turning fork gyroscope. This method was reported to achieve a rejection ratio of 390 between the antiphase modes and vibrations within a bandwidth of 0–50 kHz [82]. A dual-mass dynamically amplified gyroscope (DAG), manufactured via focused ion beam (FIB) ablation and a developed predictive tuning algorithm for identifying ablation locations, was reported to achieve an ARW of 0.021°/√h and a bias stability of 0.2°/h in permanent frequency trimming of the primary wineglass modes in planar gyroscopes with concentric ring suspensions [83].

Despite the advantages of vibration decoupling, improved vibration Q values, and anti-interference characteristics, the industrialization of dual-mass sensitive gyroscopes continues to face challenges in tuning, matching, trimming and performance optimization.

A conventional dual-mass resonator, designed with an additive oscillating structure to adjust the eigenfrequency and quality factor Q_Anchor_ related to the anchor loss by electrostatic tuning of the suspension and inner spring, was reported to achieve a Q factor increase of up to 19% with a DC bias of 15 V, whereas the resonant frequency change was only as small as 162 ppm, and the resonant frequency could be tuned as high as 7023 ppm with a Q factor change of 16% with the application of a DC bias to soften the suspension stiffness [84]. To minimize the mismatch in resonant frequencies and the Q-factors between the out-of-plane (OOP) and in-plane (IP) modes, a dual-mass structure was designed to reduce the torque applied to the supporting substrate in OOP mode. For the fabrication, two layers of Si substrate combined with Au-Au thermocompression bonding technology were used, and this structure achieved mismatches of 1.3% in resonant frequencies and 32% in Q-factors [85]. The performance of a MEMS dual-mass vibratory gyroscope, corrected via an application-specific integrated circuit (ASIC) chip with quadrature error correction, as an integrated interface circuit, as shown in Figure 3b, was demonstrated to improve in scale factor (from 43 to 40 mV/(°/s)), nonlinearity (reduced from 1326.7 ppm to 150.6 ppm), ARW (reduced from 0.25°/√h to 0.028°/√h), and bias instability (BI) (reduced from 3.25°/h to 0.29°/h) [76]. An axisymmetric dual-mass gyroscope, composed of two masses vibrating in antiphase mode, combined with electrostatic tuning for defect cancelation, electrostatic trimming for quadrature and frequency mismatch correction, and closed-loop control for controlling the drive and sense mode, was reported to achieve an ARW of 0.006°/√h, a bias instability of less than 0.012°/h, and an adjustable range of 375°/s in FTR mode [86].

#### 3.2.3. Quadruple-Mass Block Structure

A quadruple-mass gyroscope featuring four identical single-mass blocks can be considered two dual-mass-block tuning fork gyroscopes. During operation, the adjacent dual-mass blocks corresponding to the driving and detecting mode perform antiphase vibration.

Although amplitude modulation (AM) is commonly used as the displacement measurement method for most MEMS gyroscopes, it still faces challenges in improving the stability of scale factors against environmental fluctuations and manufacturing defects. Frequency modulation (FM) has been proven to be an effective solution to this issue. However, a dual-mass gyroscope with incomplete symmetry can resist only one-sided common-mode interference, making it unsuitable for FM detection. A quadrupole-mass gyroscope (QMG), as a fully symmetric structure, not only combines the advantages of single-mass block and dual-mass-block structures but also benefits from relatively simple processing technologies such as photolithography, which eliminates the need for assembly and alignment procedures.

**Figure 4:**
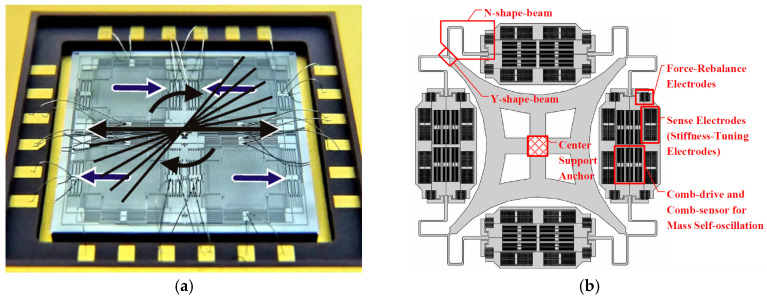

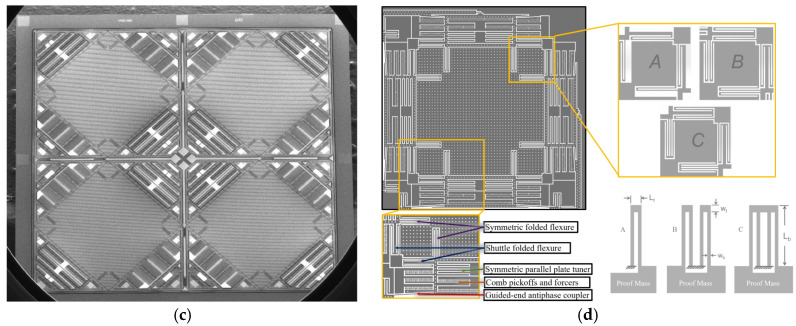
Several quadruple-mass gyroscopes: (**a**) fabricated 100 μm thick SOI QMG with an illustration of the whole-angle operation [87]; (**b**) centre support QMG [89]; (**c**) QMG with central coupling springs and four tapered levers [91]; (**d**) QMG by means of resigning the Coriolis mass folded flexures and shuttle springs [92]. Printed with permission.

As illustrated in Figure 4a, a QMG fabricated by using a 100 μm thick SOI with a symmetrical structure for whole-angle measurement, designed by the University of California, Irvine, achieved a resonant frequency of 2 kHz with a Q-factor of 1.2 million, a linear measuring range of ±450°/s, and a working BW of 100 Hz operated in whole-angle mode and the condition of mode-matching [87]. This was accomplished through the use of driving and detection closed loops on the DSP platform to ensure system stability. Similarly, a QMG, fabricated via the planar Si-on-glass (SOG) process with a device thickness of 100 micrometres, was reported by the University of Michigan and achieved an ARW of 26.4°/√h in the mode of 5 Hz with mismatched status [88].

A novel centre support quadruple mass gyroscope (CSQMG), shown in Figure 4b, integrates the advantages of tuning fork gyroscopes and a microhemisphere resonator gyroscope (HRG), achieving a Q-factor of greater than 8000, a zero-bias stability of 0.12°/h and an ARW of 0.72°/(h·√Hz) [89]. A QMG with a 2 mm × 2 mm epitaxial package, incorporating closed-loop amplitude control and an orthogonal compensation circuit, was reported to achieve a Q factor of 85,000 and an ARW of 0.0264°/√h [90].

Similarly to wine-glass vibration, a QMG (as shown in Figure 4c) designed with central coupling springs and four tapered levers for synchronizing antiphase driving motions achieved an Allan variance bias instability of 5.9°/h, an ARW of 0.28°/√h and a scale factor of 94.98 LSB/(°/s) [91]. The smallest QMG, designed with an epitaxial polysilicon thickness of 24 µm and a packaging pressure of approximately 1 mbar to obtain glassfrit-based wafer–wafer bonding within an area of 1 mm^2^, was reported to achieve an ARW of 0.006°/√h over a sensing bandwidth of 40 Hz [92]. Under mode-matching conditions, a QMG (shown in Figure 4d) designed by means of resigning Coriolis mass folded flexures and shuttle springs, linearizing the antiphase coupler spring, adopting linear combs, implementing dedicated force-balanced electrostatic frequency tuners, and having microTorr vacuum packaging was reported to achieve an ARW of 0.0005°/√h and an uncompensated bias instability of 0.08°/h [93].

Despite the advantages of QMGs in optimally rejecting acceleration and vibration effects owing to their symmetric structures along two in-plane directions, their design typically requires large-area sensors to ensure optimal suspension implementation and mode decoupling [93].

#### 3.2.4. Ring/Disc Structure

With developments in planar processing technology, ring microgyroscopes based on the Byran effect have gradually evolved to overcome inherent deficiencies caused by asymmetric structures. Ring MEMS gyroscopes are more suitable for mass manufacturing via MEMS processes. Since the 1990s, topological structures have evolved from single rings to nested ring structures and thus have formed various derivative structures, such as multiring, star, spider, honeycomb, petal ring, disc, cylinder, and even bionic nested structures.

**Figure 5:**
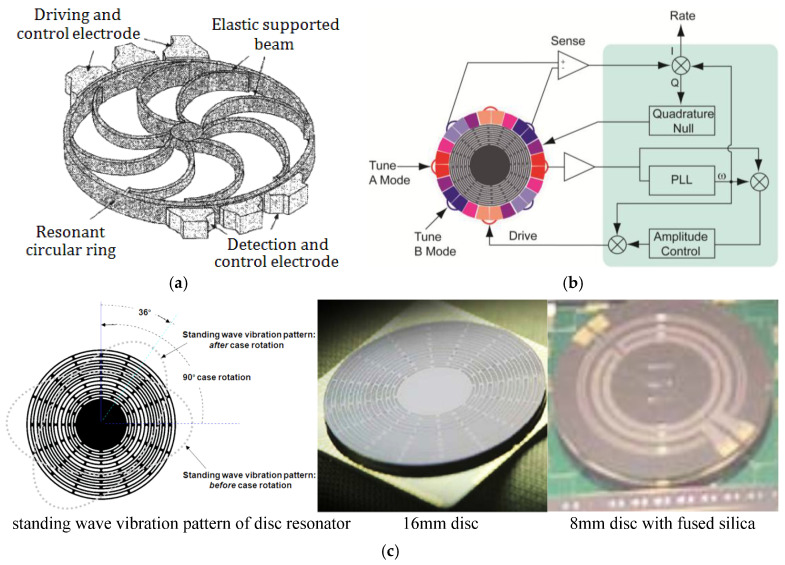
Typical ring/disc forms of resonator gyroscopes: (**a**) vibrating-ring silicon microgyroscope [94]; (**b**) diagram system of a gyroscope designed with a form of epitaxially encapsulated polysilicon disc [100]; (**c**) 8 mm fused silica disc resonator gyroscope from the Boeing Company [102]. Printed with permission.

As shown in Figure 5a, a vibrating ring gyroscope, composed of one loop, 8 elastic support beams, and driving and detection electrodes, was designed to achieve an ARW of 6°/√h, a zero-bias stability of 10°/s (temperature range: −40~+85 °C), a resolution ratio of 0.5°/s (BW:25 Hz), and a nonlinearity scale factor of less than 0.2% (measuring range: ±100°/s) [94].

A dual-axis gyroscope, fabricated via a disc-type rotor supported by an elastic beam on polycrystalline silicon, was reported to achieve a scale factor of 2 mV/(°/s), a resolution ratio of 10°/h, and an ARW of 14.4°/√h [95]. A single-crystal silicon (SCS) vibrating ring gyroscope was designed to achieve a Q-factor of 12,000, a nonlinearity of 0.02%, a scale factor of 132 mV/(°/s), and an ARW of 10.4°/(h•√Hz) [96]. A doubly decoupled vibrating wheel lateral-axis gyroscope, featuring torsional sensing comb fingers that enable differential detection of out-of-plane torsional movements while being insensitive to movements in other directions, was fabricated and tested to achieve a scale factor of 3.1 mV/(°/s), a nonlinearity of 7.68‰ within a full scale of 900°/s, and an ARW of 27°/√h [97]. A novel cylindrical rate-integrating gyroscope (CING), designed via a SOG process with a (111)-oriented Si wafer, was reported to exhibit an operating frequency of 17.9 kHz, a nominal Q-factor of 21,800, a zero-bias stability of 0.16°/s, and an ARW of 3.6°/h•√Hz) [98]. Tested with a suspended rotor spinning at a speed of 10,085 rpm, a micromachined gyroscope featuring a spinning ring-shaped rotor suspended by an electric bearing with five degrees of freedom and driven by a three-phase variable-capacitance motor and fabricated via the bulk micromachining processing technique was reported to achieve a measurement range of ±100°/s, a scale factor of 39.8 mV/(°/s), a zero-bias stability of 50.95°/h, and an ARW of 0.015°/h•√Hz [99].

As shown in Figure 5b, a gyroscope designed with an epitaxially encapsulated polysilicon disc was designed to enable a Q-factor of 50,000, a scale factor of 0.286 mV/(°/s), an ARW of 0.36°/√h, and a minimum Allan deviation of 3.29°/h [100]. In subsequent research, another gyroscope with an integrated CMOS Analog Front-End was reported to achieve a Q-factor of 2800, a scale factor of 55 μV/(°/s), an ARW of 0.048°/√h, and a zero-bias instability of 20°/h [101].

Leveraging the advantages of annular or disc structures and optimizing the design, layout, materials, and fabrication processes, an 8 mm disc resonator gyroscope (DRG), developed from a 16 mm disc (as shown in Figure 5c), was fabricated, diced and vacuum encapsulated in a ceramic leaded chip carrier (LCC) by Boeing Company. It demonstrated excellent performance, with an ARW of 0.138°/√h and zero-bias instability within 0.01°/h over a week [102].

**Figure 6:**
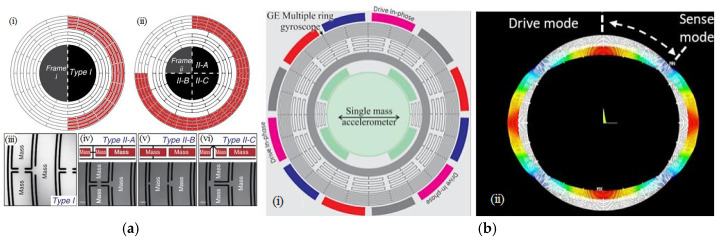
Two types of disc/ring gyroscopes: (**a**) structure of the DRG prototype [104]: Type I resonator structure with frame (i); Type II resonator structure showing one quarter of each type of DRG with different lumped mass configurations (ii); (iii–vi) close-up photographs; (**b**) MRG technology [109]: conceptual view (i) and finite element simulation to show the wineglass mode (ii). Printed with permission.

An inertial grade MEMS gyroscope, which is based on the stiffness–mass decoupling concept and technology of hanging lumped masses on the frame structure, was designed to achieve a decay time constant of 8.695 s and an ARW of 0.0009°/√h induced on the basis of Brownian noise [103]. Shortly thereafter, on the basis of this strategy and technology to add lumped masses to the frame structure to mitigate its figure of merit (FOM), a DRG prototype (shown in Figure 6a), proposed by the same research team, was reported to enable an ARW of 0.012°/√h and a bias stability of 0.08°/h [104].

On the basis of a gyroscope composed of a natural honeycomb and many interleaved hexagonal cells, to exploit the advantages of a high space utilization ratio and excellent mechanical characteristics [105], an improved stiffness–mass decoupled honeycomb-like DRG was reported to achieve a Q-factor of 171.6k, a room temperature bias instability of 0.11°/h, and an ARW of 0.083°/√h [106]. Through comprehensive optimizations of the structural parameters, lumped mass configuration, and arrangement of inner electrodes via finite element simulation, a honeycomb disc resonator gyroscope (HDRG) prototype, which was fabricated via SOI fabrication technology and tested with a closed-loop digital circuit, was reported to achieve a quality factor of 650k, a bias instability of 0.015°/h, and a scale factor nonlinearity of approximately 120 ppm in the range of ±300°/s at room temperature without any compensation [107].

A rate integrating gyroscope, designed with a micro donut-mass block as the mechanical unit for sharing a resonant frequency along the *x*-axis and *y*-axis, enabled relatively high intrinsic stability (i.e., a frequency mismatch of 80 MHz on the basis of electrostatic tuning), reducing the drift angle to 1/7 of the original value [108]. A multiring gyroscope (MRG, shown in Figure 6b), adopted with multiple concentric mass blocks together with through silicon via (TSV) technology and an etching process (with an aspect ratio of 30:1) on a SOI, was reported to achieve a Q-factor of 13,400, a bias instability of 0.01°/h, and an ARW of better than 0.003°/√h [109].

On the basis of a designed micromachined vibrating ring gyroscope (MVRG) with a ring and eight “M” type supporting beams with a central-symmetrical structure [110], subsequent feedback control via amplifiers and phase shifters enabled the gyroscope to achieve a scale factor of 8.9 mV/(°/s), nonlinearity within ±200°/s, and a resonation ratio of 0.05°/s [111]. Fabricated through a wafer-level vacuum-package process, a fully enclosed electrode MEMS DRG was reported to achieve an ARW of 0.05°/√h and bias instability of 0.42°/h within a full-scale range of ±300°/s [112].

A novel disc resonator gyroscope with a cobweb-like structure comprising 10 concentric, 16-sided spider web rings connected to a single central anchor by eight alternating spokes to achieve a stiffness–mass decoupling design from a traditional ring-like dis resonator gyroscope to a cobweb-like disc resonator gyroscope was reported to enable a scale factor of 98.1 mV/(°/s), an ARW of 0.004°/√h and zero-bias instability of 0.87°/h [24]. A hermetically sealed disc acoustic gyroscope, operated in elliptic mode with an attenuation coefficient of *n* = 3 at approximately 3 MHz as the mode and a high Q-factor of 165,000, was reported to achieve a bias instability of 0.85°/h and an ARW of 0.004°/√h [113].

A radially pleated disc resonator, composed of concentrically nested radially pleated rings, was reported to achieve an angular gain factor uncertainty of 1% and a measurement relative deviation of no more than 0.68% for the full range output of ±1000°/s under a clockwise quadrature frequency-modulation (QFM) system at 4.27 Hz [114]. A flower-like disc resonator, made up of concentric meandering-shaped rings interconnected by straight beams, was reported to decrease the resonant frequency and frequency split by 39.1% and 70.2%, respectively, and the Q and decay time improved by 63.8% and 172%, respectively, compared with those of traditional ring-like disc resonators [115]. A single-crystal lithium niobate (LN) wafer (i.e., 155°-Y-cut LN) with uniform characteristics of elastic compliance in any in-plane direction was used to design and fabricate a 25.8 mm diameter, 330 μm thick disc resonator with 16 electrodes on each side via photolithography and grinding processes. The designed MEMS DRG was reported to oscillate in two wine–glass modes with a small frequency split of 0.7% at 95 kHz and achieve a scale factor of 0.35 μV/(°/s) without any preamplifier [116].

Micro HRGs and ring/disc resonator gyroscopes share many structural connections. Generally, all types of ring/disc microgyroscopes are the result of structural evolution from a three-dimensional structure (such as hemispherical resonator microgyros) to a two-dimensional junction. Although both methods exhibit excellent performance in terms of ARW, zero-bias instability, etc., micro HRGs have superior qualities in certain aspects.

Table 4 provides a comparative summary of the key performance indices for dual-mass, quadruple-mass, and disc/ring MEMS gyroscopes, highlighting their representative characteristics and application relevance.

#### 3.2.5. Hemisphere Structure

The basic working principle of hemispherical gyroscopes involves measuring angular velocity via the inertial effect of elastic standing waves [118]. Micro-HRGs and ring/disc micromachined gyroscopes typically operate at *n* = 2 (i.e., wineglass mode) or *n* = 3 (i.e., cloverleaf mode). For measurement tasks, HRGs can be operated in either whole-angle mode or force-to-rebalance mode. The former is suitable for high-stability measurements of high-speed rotating objects, whereas the latter is ideal for high-accuracy measurements of low-speed rotating objects [119].

**Figure 7:**
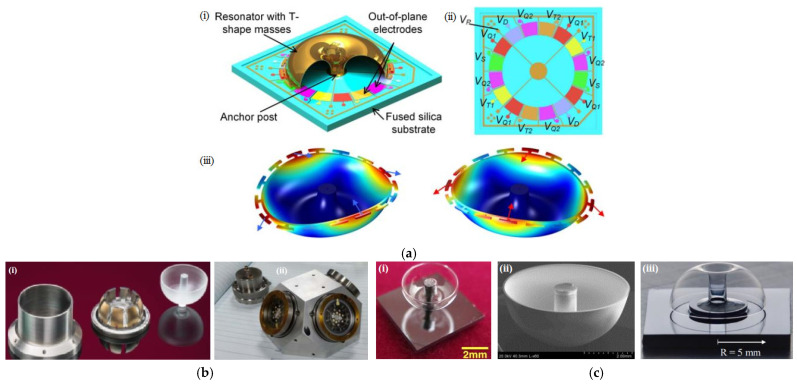
Several typical HRG/birdbath resonator gyroscopes: (**a**) architecture of MSRG [120]—schematic view of the device (i), electrode configuration (ii) and wineglass modes (iii); (**b**) Gen-2 milli-HRG [122]—key components (i) and mHRG ISA Demonstrator with Gen 2 Mhrg gyros (ii); (**c**) Birdbath resonator gyros developed by the University of Michigan—made by assembling released birdbath shell (radius: 2.5 mm, height: 1.8 mm) on Si substrate (i) [131], fused shell attached with fused silica solid stem (ii) [132] and attached to silicon substrates with glass frit (iii) [133]. Printed with permission.

Designed with a suspended resonator and a fused silica substrate, which is defined as out-of-plane electrodes, a microshell resonator gyroscope (MSRG) (shown in Figure 7a) with sixteen T-shaped masses, which are taken as the shell edge and connected with the shell by a thin support beam, was reported to enable a scale factor of 0.107 V/(°/s), an ARW of 0.099°/√h and a bias instability of 0.46°/h under the force—rebalancing mode based on the driving and sensing mode of *n* = 2 [120].

Designed by the University of Michigan, a fused-silica microprecision shell integrating (PSI) gyroscope with a diameter of 10 mm operating in force-rebalanced mode was reported to achieve a Q factor of 5.2 million under vacuum packaging conditions, a short-term in-run bias instability of 0.0014°/h and an ARW of 0.00016°/√h [121]. As a typical representative of research on high-performance hemisphere gyres and achieving small HRGs, Northrop Grumman Co. has been engaging in gyroscope research since the first HRG prototype with a wineglass shape was designed in 1965. Under the dual gyro self-calibration tests, a type of Gen-2 milli-HRG (mHRG) (shown in Figure 7b) with a simplified design containing 10-fold fewer parts than the space gyro was reported to achieve a bias stability of 0.0005°/h. Another mHRG configuration (130Pi) was reported to achieve a bias stability of 0.00015°/h [122]. Without self-calibration, these two types of individual gyroscopes can achieve ARWs of 0.0007°/√h and 0.00010°/√h, respectively. Similarly, Sagem Company has spent two decades researching 3 generations of HRG products and developing HRG technology to the world’s leading level. As a representative of third-generation products, a type of HRG Crystal^TM^ Dualcore was reported to achieve an ARW of 0.0002°/√h and a zero-bias stability of 0.00007°/h [123].

Typically, the measurement accuracy of a hemispherical resonator is not limited by size, allowing for direct angle measurements with a large dynamic range. This characteristic has inspired the development of microhemispherical shell resonators. Typically, microhemisphere gyrostructures can be divided into surface microhemisphere gyrostructures (achieved by depositing a thin film on a predefined die) and bulk microhemisphere gyrostructures fabricated through plastic deformation or ultrasonic processes [124]. In particular, research on microhemispherical gyroscopes using thin film deposition on different materials, such as polysilicon [125], diamond [126], alumina [127] and silica [128], has rapidly developed. Additionally, integrating silicon photonic integrated circuits with MEMS has effectively improved the design structure and performance. A novel optomechanical gyroscope based on a microhemispherical shell resonator integrated with optical ring cavity resonators, with high-Q optical ring resonators coupled via evanescent fields from an on-chip silicon waveguide as the basic building block, was demonstrated to attain a scale factor of 77.9 mV/(°/s) and a total ARW of 0.0662°/√h for a microhemispherical shell mass of 212 ng at an input laser power of 5 mW through numerical simulation [129].

With the development of analytical models for predicting the shape of glass shells, a method for fabricating a microhemisphere resonator on silicon substrates via glass blowing was reported to achieve an internal surface roughness of 9 nm and an outer surface roughness of 2 nm [130]. A birdbath resonator gyroscope (BRG) (shown in Figure 7c(i)) designed for batch-level microfabrication via blow torching was reported to enable a Q-factor of 294,450 and a minimum normalized frequency split of 0.24% in the mode of *n* = 2 [131]. On the basis of the above preliminary design and processing technology, an improved design (shown in Figure 7c(ii)), which is fabricated by flowing the shell and welding it to a solid rod at a controlled location in one step and in a single mold, was reported to enable a Q-factor of 2.55 million, a ring-down time of 35.9 s, and a resonator frequency of 22.6 kHz [132]. Under the force rebalancing mode in 2017, this design achieved an ARW of 0.00126°/√h and a zero-bias instability of 0.0391°/h. Furthermore, by utilizing a graphite mold to form fused silica shell resonators and reflowing molding to smooth the suspended fused silica surfaces to an average roughness of 1.8 Å, a birdbath shell resonator gyroscope with a 5.0 mm radius (as shown in Figure 7c (iii)) was reported to achieve a Q-factor of 4.45 million and a ring-down time of 259.0 s [133].

By adopting the same micro glassblowing and fabricating process described in [120], a microhemispherical resonator featuring teeth-like tines along the perimeter, fabricated from ultrafast laser ablation, was reported to enable a Q-factor of 1.18 million, a full-scale range of ±200°/s, a bias instability of 0.133°/h at room temperature without compensation, and a fabricated frequency split of 17.8 Hz, which was subsequently reduced below 70 MHz after mechanical trimming [134]. A navigation-grade birdbath resonator gyroscope with a diameter of 5 mm and a wineglass mode resonance of *n* = 2 was reported to enable a Q factor of 1.54 million and zero-bias stability of 0.0103°/h [135].

Additionally, a chemical foaming process (CFP) for the fabrication of three-dimensional wineglass shell structures was experimentally demonstrated to obtain stems with different shapes and self-formations with extremely smooth surfaces (0.1 ppm relative roughness, 0.332 nm Ra) [136].

From the above introductions, it is evident that the development of diverse gyroscope forms and structures has yielded valuable technological insights and applications, significantly advancing this field. Some conclusions are given as follows:

(i) QMGs, featuring fully symmetrical structures and two degenerate operating modes, represent advanced mass–block gyroscopes capable of full-angle operation and exhibit superior performance among planar MEMSs.

(ii) Ring/disc-shaped HRGs, resulting from structural innovations, offer relatively easy-to-achieve symmetrical structures and excellent performance, making them among the most attractive MEMS gyroscopes.

(iii) HRGs, despite their complex 3D structures imposing strict design and manufacturing requirements, hold great potential for achieving high precision, reliability, and widespread use in long-term positioning and inertial navigation systems. Research trends indicate a move toward miniaturization in HRGs while maintaining the key performance attributes of the harmonic oscillator.

## 4. Impacts of Design, Fabrication and Factors on Performance

### 4.1. Performacne Impacts from Pespective of Design Types

In terms of structural design, a tri-axial surface-micromachined MEMS vibrating gyroscope using a rotational comb drive as both a capacitive sensor and an actuator eliminates the need for magnets and metal casings, facilitating the development of a true monolithic MEMS-IC electronic system [137]. Additionally, the use of mechanical amplification structures in MEMS tuning fork gyroscopes significantly enhances mechanical sensitivity by increasing the displacement caused by Coriolis forces [138].

Manufacturing costs and integration are essential considerations. The application of 3D wafer-level packaging technology in high-Q dual-mass MEMS tuning fork gyroscopes reduces thermal elastic damping and anchor losses, achieving a high Q-factor while enabling wide measurement ranges and high resolution [139]. Additionally, the development of a Y-axis MEMS gyroscope based on CMOS-MEMS technology, optimized via a multi-objective genetic algorithm for structural dimensioning, provides a solid foundation for the integration of gyroscope structures and circuits [140].

In Table 5, several performances of typical architectural solutions are summarized. 

**Figure 8:**
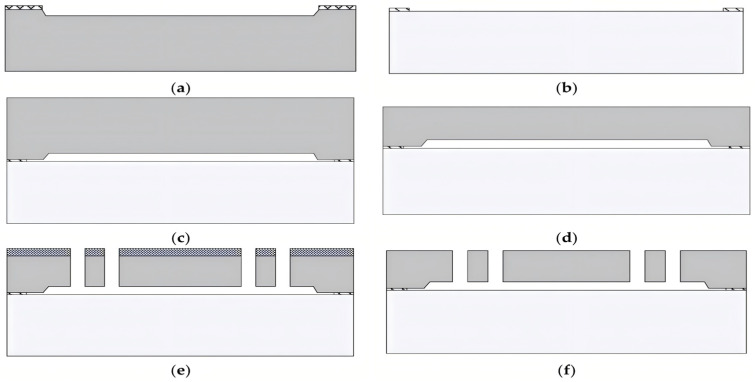
Typical steps of SOG fabrication process [146]: (**a**) Formed anchors on silicon substrate; (**b**) Pattern of Cr/Au at the glass substrate; (**c**) Anodic bonding of silicon and glass substrates; (**d**) CMP of the silicon; (**e**) Pattern of the photoresist etch mask for DRIE and thoroughly etched silicon substrate; and, (**f**) Remove photoresist etch mask. Printed with permission.

Moreover, the manufacturing process is crucial in determining the performance of MEMS gyroscopes. Figure 8 show the case of SOG fabrication process, which mainly include wet etching or drying etching silicon, anodic bonding of silicon and glass, chemical mechanical polishing (CMP) the silicon to obtain the certain thickness, and Deep reactive-ion etching (DRIE) the silicon to form the designed structures [146]. Such design and fabrication significantly enhances the reliability and precision of the gyroscope. 

### 4.2. Performacne Impacts Caused by Certain Factors

In addition to the comparison of different design solutions mentioned above, some design solutions for MEMS gyroscopes suitable for standard consumer applications are further explored on the basis of considering critical factors such as shock resistance, precision, and sensitivity, to optimize their performance in real-world environments.

#### 4.2.1. Environmental Sensitivity

Precision and sensitivity are the crucial factors in the design process. Table 6 summary several impacts caused by environmental sensitivity. These metrics not only highlight the advantages and limitations of each design but also serve as a guide for optimizing MEMS gyroscopes in real-world applications.

**Figure 9:**
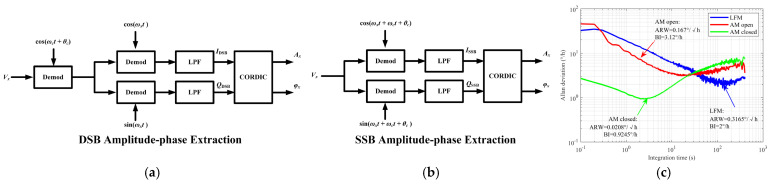
Impact of modulation techniques on gyroscope performance [48]: (**a**) Amplitude and phase extractions for double-sideband (DSB) modulation. (**b**) Amplitude and phase extractions for single-band (SSB) modulation. (**c**) A 1 h Allan deviation of the gyroscope operating in linear frequency modulation (LFM) mode, amplitude modulation (AM) open-loop mode, and AM closed-loop mode. Printed with permission.

In order to address the challenges brought by the impact of environmental sensitivity on performance, some measurements were adopted to enhance the performance. Two types of modulation techniques (amplitude-phase extraction) together with close-loop control were adopted to achieve ARW 0.0208°/√h and BI (Bias Instability) of 0.9245°/h shown in Figure 9. MEMS gyroscopes driven electrothermally with multiple degrees-of-freedom (DOF) actuation have demonstrated superior performance by operating within flat regions of drive mode, thereby reducing the impact of environmental and manufacturing variations on device performance [148]. 

#### 4.2.2. Sock Resistance

Shock resistance is a key factor in MEMS gyroscope design for consumer applications. Recent developments in MEMS gyroscope designs have significantly increased their shock resistance. By implementing a dual-mass structure and a two-stage elastic stopper mechanism, manufactured via the silicon-glass deep etching (DDSOG) technique, the shock resistance of the gyroscope was significantly enhanced. The experimental results show that this design improved the shock resistance of the gyroscope by 1900 g and 1500 g in the x and y directions, respectively. The prototype demonstrated shock resistance exceeding 10,000 g in the *x*-, *y*-, and *z*-axes and could recover normal output within approximately 2 s after being subjected to shock [56]. By using Single Degree-of-Freedom and Two Degree-of-Freedom models, deducing the U-folded beam stiffness and maximum positive stress to evaluate the shock resistance of the silicon beam, another study presented a shock-protected structural design capable of improving shock resistance approximately 13-fold across a wide temperature range from −40 °C to 80 °C [149]. These design strategies not only improve the mechanical sensitivity but also enhance the device’s stability under extreme conditions.

The vibration significantly impacts the parameter stability of MEMS gyroscopes. To address this, researchers have developed an accelerated degradation model to assess the impact of vibration on a gyroscope’s zero bias and scale factor. The experimental results indicate that as the vibration stress level increases, the failure time of the MEMS gyroscope decreases, and the failure rate increases, particularly with significant changes in the zero-bias parameter. This model effectively characterizes the reliability changes in MEMS gyroscopes under vibration, providing valuable insights for practical applications [150].

#### 4.2.3. Thermal Stress and Temperature Drift

Thermal stress is one of the key factors affecting the performance of MEMS gyro-scopes. Research has shown that by designing novel cantilever plate structures, the influence of thermal stress on MEMS gyroscope performance can be effectively mitigated, leading to significant performance improvements. Specifically, after the cantilever plate structure was integrated, the stress on the MEMS gyroscope was reduced 346-fold, the average capacitance gap error was reduced 36-fold, the frequency variation decreased by 28.6%, and the bias stability improved approximately 2-fold [57].

The influence of temperature drift on the performance of a gyroscope should never be underestimated. Recent studies have demonstrated that the combination of mode-reversal techniques and multiple regression compensation methods can effectively suppress temperature drift, thereby improving the zero-bias stability of gyroscopes [151]. Furthermore, the implementation of temperature compensation circuits and real-time self-compensation algorithms has significantly reduced bias errors across a wide temperature range, enhancing gyroscope stability under varying environmental conditions [152].

#### 4.2.4. Other Optimization Technologies

In terms of design optimization, machine learning-driven approaches have shown great promise in discovering high-performance MEMS disc resonator gyroscope topologies. By combining deep reinforcement learning and convolutional neural networks, researchers have been able to identify novel structural topologies that significantly outperform traditional designs in terms of performance [153]. This innovative design approach offers new avenues for future MEMS gyroscope development, facilitating the rapid discovery of advanced structural configurations.

Another breakthrough has been the integration of MEMS gyroscope arrays. By optimizing the performance through Kalman filtering, multiple identical MEMS gyroscopes can be combined into a virtual gyroscope, offering superior performance compared with individual sensors [154]. This approach not only improves measurement accuracy but also provides a viable path for low-cost MEMS sensors to be applied in high-precision applications, further demonstrating the potential of MEMS gyroscopes in advanced applications.

In addition, based on deep learning, the output model of MEMS IMU (inertial measurement units) gyroscope is constructed, using the temporal convolutional network. The attitude and position accuracy obtained by the inertial navigation solution were effectively and accurately compensated by using regression to achieve error compensation based on error features obtained from the data in the past [155].

### 4.3. Fabrication Processes on the Impact of Permormance

Different fabrication processes of MEMS gyroscope significantly impact their performances, which of them focus on surface quality, structural integrity, and Q-factor optimization. The details of different process types, corresponding to key parameters, performance outcomes, and limitations are summarized in Table 7, Table 8, Table 9, Table 10, Table 11 and Table 12, providing valuable insights for choosing the most effective manufacturing methods.

Besides impacts mentioned above, the fabrication process also has other profound impacts on the performance of gyroscopes. From mechanical properties to stability, precision and reliability, every aspect is constrained by the fabrication process.

### 4.4. Brief Summary

In line with the comparison and analysis viewed form the aforementioned design and fabrication technologies of MEMS gyroscopes, significant advancements have been made in recent years, particularly in terms of enhancing shock resistance, temperature drift compensation, and machine learning-driven design optimization. These advancements not only optimize performance and reliability, but also provide stronger support for applications in complex environments. Furthermore, these methods and strategys provide a strong theoretical foundation and technical support for the future development of MEMS gyroscopes, with a purpose of focusing on improving performance and reliability, particularly for use in demanding environments.

The design of a versatile MEMS gyroscope for consumer applications requires a comprehensive approach that balances shock resistance, precision, sensitivity, manufacturing cost, and integration. By leveraging advanced structural designs and manufacturing technologies, high-performance MEMS gyroscopes can be realized for consumer-level applications. MEMS gyroscopes have found widespread applications in the consumer electronics, automotive, industrial, and military sectors owing to their low power consumption, ease of integration, and cost-effectiveness. However, reliability under extreme conditions remains a major challenge for MEMS gyroscopes [160].

Totally, the performance optimization of MEMS gyroscopes in terms of thermal stress, shock resistance, and vibrational environments remains a key focus of current research. Through further structural design optimization and improvements in reliability assessment methods, these technical bottlenecks can be overcome to lay a solid foundation for the widespread application of MEMS gyroscopes. Future research should continue to address these aspects to advance MEMS gyroscope technology.

## 5. Features and Challenges of Gyroscope Technology

In this section, the typical characteristics and challenges of gyroscope technologies are summarized in the following two parts.

### 5.1. Typical Characteristics of Gyroscope Research

Gyroscope research has three prominent characteristics: interdisciplinary characteristics, the statistical nature of the results, and the correlation between compensation and calibration.

#### 5.1.1. Multidisciplinary Integration

Taken as highly sensitive sensors for angle or angular velocity measurement, gyroscopes are the product of a complex system involving multiple disciplines, such as mathematics, mechanics, materials science, electronics, control theory and reliability engineering. Without exception, the research and development of gyroscopes, starting from initial design to final calibration and commercial application, require a comprehensive understanding of various principles, methods and techniques across these disciplines.

(i) Common materials used in gyroscope research include single-crystal polysilicon, silica, alumina and even diamond.

(ii) Depending on specific requirements, fabrication processing may involve several physical techniques, such as thin-film deposition, photoetching technology, SOI, spin-on-glass, glass blowing, femtosecond ablation, and new material generation (synthesis) technologies, such as graphite–selenium, carbon fibre polymer (CFP), and lithium niobate.

(iii) Innovations in microstructure have significantly improved gyroscope performance, as detailed below:

(1) Concentrated mass structures: These structures, including amplitude-based dual-mass structures, dynamic amplifying dual-mass, and dynamic balancing dual-mass resonators, generally adopt tuning fork designs with various driving/detection and connecting beams (e.g., straight, U-shaped, and folded). Variable capacitance comb tooth structures are commonly used for generating driving/detection forces. With similar driving/detection and connecting structures, quadruple-mass structures involving dual-mass structures are specially designed (i.e., series mutual coupling or parallel coupling) to achieve better performance.

(2) Ring/disc structures: Advanced planar processing technology has enabled ring/disc microgyroscopes to overcome inherent asymmetric issues. Enhanced forms such as honeycomb-like DRGs, MRGs, and spider web rings can achieve high performance, approaching quasinavigation accuracy in terms of the Q-factor, ARW, and zero-bias stability.

(3) mHRGs: These vacuum-encapsulated wineglass/microbasin-shaped resonators utilize elastic standing waves for high-stability/accuracy measurements, making them suitable for deep space navigation and positioning because of their excellent performance.

(iv) Electronics and control technology: Signal detection, processing, mode matching [80,161,162,163,164], closed-loop control [26,110,111,165,166], and system calibration and compensation technologies [167,168] are crucial for ensuring that gyroscopes are in a normal operation status. For example, the use of phase-locked loop (PLL) technology combined with automatic gain control (AGC) for closed-loop driving and detection ensures stable system operation, reduces common-mode interference and improves the SNR. The digital phase-locked loop technology used in driving loops is commonly used to compensate for the detection force to correct the orthogonal error, and the use of PID control in detection loops ensures sufficient system stability margins and steady-state accuracy. Generally, the electronic control architecture of a system is related to the working mode of the MEMS gyroscope, i.e., rate measurement and whole-angle measurement.

(1) Rate measurement gyroscopes can be operated in either open-loop or force-to-rebalancing (FTR) mode, whose typical technologies include multiparameter fusion for compensation, quadrature error elimination via a self-synchronous sigma-delta modulator, adaptive online self-calibration, correction of the relative phase drift between the modulation and demodulation references, phase compensation for zero-bias drift, stochastic error compensation via an adaptive Kalman filter, etc.

(2) Known as a type of RIG, the whole-angle measurement gyroscope, which is generally implemented by a closed-loop control system for maintaining constant amplitude, suppressing orthogonal errors, and tracking/controlling the directional precession mode without interfering with the measured precession mode. Typical electronic and control technologies employed in RIG include interface design to ensure that the bulk acoustic wave (BAW) maintains automatic pattern matching, automatic calibration of asymmetrical microcontrollers, a triangular variable area capacitor for amplifying or attenuating linear parameters, and self-calibration.

Furthermore, from a quantitative inference and deduction perspective, modelling of the gyroscope system; stress modelling and analysis of each connection structure; and reliability design and experimentation [41,169,170,171] rely heavily on mathematical tools such as linear/nonlinear differential equations and statistical inference for modelling, measurement, prediction, and evaluation of the system.

#### 5.1.2. Statistical Properties of Results

As described in Section 3, gyroscopes exhibit markedly different performance characteristics. Generally, performance metrics such as the Q-factor, ARW and zero-bias stability of hemisphere or ring/disc gyroscopes are superior to those of mass-block gyroscopes. However, this conclusion is not absolute because numerous uncertain factors influence the output of the gyroscope. These uncertainties are inherently introduced into the results. From both macroscopic and microscopic perspectives, two explanations can be provided as follows:

(i) Technological differences across disciplines can lead to significant variations in performance. Specifically, the performance of a gyroscope is largely determined by the structure design and fabrication processes. For example, dual-mass, quadruple-mass, ring/disc, and hemispherical gyroscopes are required with strict structural symmetry. Even with perfect design, environmental vibrations, temperature and humidity changes, parameter drift, and fabrication errors can cause substantial performance variation. Consequently, key performance indicators such as ARW and zero-bias stability (or Allan variance) are often evaluated statistically.

(ii) To achieve adaptability across a wide range of environments, low power consumption, low cost, lightweight design, small size, high performance, and high reliability, researchers have continuously strived to meet these goals since the inception of MEMS gyroscopes. However, as the size (mass) decreases, the overall performance of gyroscopes deviates from macroscopic theories, behaviors and physical characteristics related to materials and structures. In different environments and operating conditions, mechanical parameters inevitably change due to so-called scale effects, surface effects and contact effects, which diverge from macroscopic mechanics and physical laws.

(1) First, when the gyroscope scale is less than 1 mm, the electrostatic force becomes relatively stronger than gravity. On the one hand, using electrostatic force as the driving force for micromechanical structures limits the achievable force and displacement due to constraints on power consumption, voltage or volume, as silicon-based structures cannot withstand large bending deformation. On the other hand, driving and detection methods must adapt to the reduced structural size. This means that a method/technology with sufficiently high sensitivity is needed to detect/discern small forces, displacements and their corresponding changes. These limitations caused by scale effects reduce the accuracy of the system’s mathematical model, potentially leading to performance discrepancies.

(2) In the microscale state, surface forces such as electrostatic forces, surface tension, van der Waals forces generated from instantaneous polarization from atoms and molecules under quantum mechanics, and the Casimir force, which is influenced by laminar flow, turbulence or other random factors, introduce unpredictable behavior. In this case, accurate prediction of the action of a gyroscope becomes challenging.

(3) When the gyroscope scale is reduced to a certain degree, the surface energy and derived surface force lead to microscopic mechanical phenomena such as adhesion, contact and deformation on the solid surface. Therefore, traditional continuum mechanics methods are insufficient for addressing these issues. Moreover, when surface adhesion exceeds the resilience of the microstructure, the resulting known friction problem complicates the surface micromechanical model more than the bulk model does.

Compared with conventional gyroscopes, MEMS gyroscopes are generally significantly smaller in scale, mass, and volume. Consequently, the working mechanism and modelling are highly complex, leading to uncertain operational outcomes. This uncertainty underpins the statistical properties observed in MEMS gyroscopes.

#### 5.1.3. Error and Corresponding Compensation

As previously discussed, the performance of a gyroscope is critically influenced by the processing technology and topological structure. However, design and fabrication limitations, along with various environmental interferences, often result in outputs that do not accurately reflect the desired values. These errors, arising from both internal structural design and external loading stresses, significantly reduce the accuracy of the gyroscope. Errors can be categorized into deterministic or stochastic cases, necessitating two primary compensation/calibration methods: analytical and experimental approaches.

(i) Internal structural design-based error and compensation

For single-mass-block structures, external shocks or vibrations that align with the Coriolis force cannot be distinguished, leading to measurement inaccuracies. The coupled damping and stiffness forces further contribute to the output errors. These factors primarily cause low sensitivity, poor signal-to-noise ratios and high complexity in structural design and fabrication.

For dual-mass-block structures, orthogonal coupling errors are mainly determined by the asymmetry and coupling stiffness coefficient of elastic beams. Eliminating or reducing these errors is crucial for improving the performance of MEMS gyroscopes. Structural decoupling and electrical decoupling technology are adopted to reduce the orthogonal coupling error. The former is achieved by reducing the stiffness coupling coefficient of the elastic beam, whereas the latter is achieved by designing the orthogonal coupling elimination electrode [172].

Additionally, machining errors such as lithography, etching, bonding, and residual stress introduce in-phase and antiphase modes into the elastic beam coupling structure, leading to so-called in-phase–antiphase coupling during operation. Similarly, structural coupling, a method that increases the stiffness difference ratio and width of the elastic beam, and electrical decoupling, a method of designing stiffness matches on the basis of the electrostatic negative stiffness effect, are used to suppress or eliminate in-phase and antiphase coupling.

Quadruple-mass-block structures combine the advantages of single-mass and dual-mass-block design structures while avoiding their drawbacks. Symmetrical differential outputs in both the X- and Y-directions can offset environmental influences such as temperature and pressure on overall structural modes, enhancing the stability of the system.

(ii) External loading stresses and uncertain interference-based error and compensation

Improving the precision of gyroscopes typically involves anti-interference hardware design and software-based error compensation.

Hardware design includes the creation of anti-interference structures and compensation loops to increase the accuracy of specific MEMSs. For example, system phase noise analysis and modelling of noise for the QMG of a MEMS led to a compensation circuit using a two-stage capacitive amplifier instead of a transimpedance amplifier, reducing the signal noise and demodulation noise [173]. Other hardware compensation methods include adding a coupling stiffness correction circuit and an interface application-specific integrated circuit (ASIC) chip with quadrature error correction on a dual-mass vibratory MEMS gyroscope to improve the performance of the scale factor, ARW and bias instability [76]; configuring the drive mode toward the damping axis on the mode-matched honeycomb disc resonator gyroscope to improve bias instability and RMS [174]; adopting a real-time automatic mode-matching method that leverages the phase-shifted features of virtual Coriolis forces [175]; introducing parasitic-capacitance coupling errors into the traditional gyroscope model; and analyzing the scale factor nonlinearity induced by coupling errors to enable calibration of coupling errors through the least square method based on measurements of the amplitude of the driving excitation signal and axis tuning voltage to improve the nonlinearity of the scale-shift factor [176]; and presenting an identification method and correction of phase errors by adopting a phase shift module on the basis of the additional angle drift (AAD) of the whole-angle MSRG to improve its performance in terms of scale-factor nonlinearity and bias instability [177]. However, owing to machine technology and small-size effects, the costs of hardware compensation are extremely high, and the desired results are often difficult to achieve in actual applications.

For software compensation, it is crucial to analyze the error characteristics of the target gyroscope before application, study the internal and external factors of error and their transmission characteristics, construct an appropriate model for the project, and then design corresponding error software compensation algorithms to improve measurement accuracy.

Generally, the error compensation of a MEMS gyroscope involves three aspects: error modelling, error propagation and error compensation techniques.

Deterministic error models include static terms caused by external input acceleration and dynamic terms caused by angular velocity and angular acceleration generated from the rotation of the carrier. In addition, modelling and compensating for errors caused by temperature and vibration stress are key research areas. Owing to the uncertainty of environmental factors, the random drift of a gyroscope is often a nonlinear, nonstationary and slow time-varying random process. The Allan variance method, which serves as a basis for the modelling of random errors in gyroscopes, is commonly used for analyzing and representing noise characteristics, such as ARW, zero-bias stability, rate quantization noise, and Markov noise.

Random drift modelling and compensation are typically based on IEEE standard equations. The compensation methods include batch and automatic processing of specific gyroscope parameters and multiparameter time-varying compensation. Error identification forms the basis of compensation. From the perspective of error properties, errors in MEMS gyroscopes generally consist of trend items and statistical items, such as zero-bias stability and ARW. The essence of trend item compensation consists of solving a polynomial linear (nonlinear) regression equation on the basis of experimental data to obtain the coefficient of the polynomial model, whereas statistical item compensation relies on regression analysis and frequency domain analysis (e.g., Fourier analysis, spectrum/power spectral density analysis) to identify zero bias and establish various compensation models (e.g., ARMA model, neural network, support vector machine, wavelet analysis, fuzzy adaptive compensation, etc.) on the basis of Allan variance technology. For example, a parallel processing algorithm integrating permutation entropy (PE), local characteristic-scale decomposition (LCD) and an adaptive network-based fuzzy inference system (ANFIS) was adopted to compensate for temperature-induced output drift in a dual-mass MEMS gyroscope [178].

### 5.2. Challenges of Gyroscopes Technology

Significant progress has been made in gyroscope technology, with numerous contributions to theories and applications. To date, gyroscope technology remains one of the most active and successful methods for inertial measurement (IM) and inertial navigation (IN). However, several challenges persist.

#### 5.2.1. Challenge in Error Reduction Methods

Owing to the limitations of structural design, which are constrained by machining and fabrication processes, all types of irrational errors, such as orthogonal errors and common mode interference from mechanical coupling, reduce the measurement accuracy of gyroscopes. Although some active measures, such as mechanism optimization, improved processing technology, closed-loop driving, and open-loop detection, can be used to increase the detection sensitivity, they also increase the complexity of the system, cost, and reliability requirements. The continuous exploration of error reduction methods remains an ongoing endeavor.

#### 5.2.2. Challenge from the Enhancement of Mechanical Sensitivity

The mechanical sensitivity of a resonant MEMS gyroscope is determined by the amplitude of the driving signal and the difference in frequency between the driving frequency and detection frequency (i.e., the mechanical sensitivity is proportional to the former and inversely proportional to the latter). On the one hand, the input signal, which is detected through demodulation of minute capacitance changes from extremely weak analog signals, is susceptible to noise interference. On the other hand, the accuracy of mode matching between different driving modes and detection modes is constrained by the quality of the mechanical or electrostatic trimming and adjustment. The former is influenced by structural design, material selection, and fabrication processes, whereas the latter inevitably faces the environmental factors and complexity of calibration units, both of which determine device stability. In summary, enhancing the accuracy of mode matching to improve the mechanical sensitivity of MEMS gyroscopes is a critical issue that must be addressed.

#### 5.2.3. Challenge in Mathematical Modelling of a System

The foundation of characterizing and analyzing the behavior of tuned gyroscopes consists of establishing a dynamic model of the system. However, establishing and solving such models is complex and challenging for the following two reasons:

(i) In the microscale case, the macroscopic dynamics law is no longer applicable because of the dominant role of the microforces.

(ii) Various time-varying internal and external factors result in variable coefficient partial differential equations for mathematical models.

Given these challenges, obtaining a precise mathematical model or stable analytical solution of the system is difficult, further complicating the understanding of the actual behavior of MEMS gyroscope systems.

#### 5.2.4. Challenge in Machine, Levelling and Excitation Technologies

Despite the high research value and potential applications of HRG technologies due to their excellent performance, this does not mean that all the problems associated with them have been solved. In fact, noteworthy challenges such as machining, levelling, and excitation need to be solved well.

(i) The machining accuracy of the harmonic oscillator, which is taken as a core component of the HRG, poses the first challenge. First, fused quartz, which was chosen for its dimensional stability, is hard and brittle, making processing extremely difficult. In addition, the processing accuracy significantly impacts the performance because inherent processing errors, such as circular errors in the inner and outer circles, surface accuracy, coaxial error, and surface roughness, affect the uniformity of the wall thickness of the harmonic oscillator, resulting in undesirable vibration performance.

For example, although the surface physical dimensional accuracy can reach micron levels and the surface finish can reach an order of magnitude of 10^−8^, the split frequency difference in vibration typically ranges from 0.1 Hz to 0.5 Hz, an inertial navigation gyroscope requires a split frequency difference of less than 0.01 Hz, and a high-accuracy HRG may require a split frequency difference of approximately 0.001 Hz. Therefore, reducing machining errors caused by the machining process should be a primary concern.

(ii) Because of the inevitable presence of machining errors during processing, levelling technologies, including mechanical mass removal, laser processing, chemical processes and ion beam levelling, are employed to ensure that the oscillator has full directional mass symmetry. In practice, the ion beam levelling method, which uses ion etching/film coating technology with minimal impact on spherical shell material structures, is generally preferred over other technologies that can degrade the acoustic properties of HRGs. For example, mass removal via chemical processing levelling technology may increase the Q-factors while increasing the difference in frequency. The theory, method, and design of levelling technologies are highly practical and closely related to engineering experience.

(iii) Selecting an appropriate excitation mode is crucial for ensuring the accuracy of the HRG. To prevent interference with the natural resonant frequency and avoid introducing extraneous noise sources, electrostatic excitation is typically preferred over piezoelectric and magnetic excitation. However, this approach still faces several challenges:

(1) A sophisticated closed-loop excitation circuit along with its associated control system and ultrasensitive electrostatic detection method for weak vibration signals must be meticulously designed.

(2) To ensure effective excitation and enhance the sensitivity of the HRG, higher excitation voltages are needed. This necessitates stringent requirements for structural machining, assembly, and maintaining a high Q value of the oscillator, as excessively high voltages can lead to dielectric breakdown in narrow gaps.

(3) The excitation electrode system must be elaborately designed to provide a full range of excitation, enabling precise control over the nodes/bellies positions of the vibration. Additionally, high-precision monitoring of the instantaneous position of standing wave nodes/bellies is essential for accurately correlating precession angles with signals from fixed electrodes.

Moreover, challenges related to error modelling, vacuum sealing, and reliability verification in HRGs can never be underestimated.

### 5.3. Suggestions for Future Research Directions

First, thermal stress management plays a crucial role in the future performance enhancement of MEMS gyroscopes. Research has shown that thermal stress is one of the key factors affecting the temperature-dependent performance of MEMS gyroscopes. By designing novel cantilever plate structures, the thermal stress can be significantly reduced, thus improving the gyroscope’s performance. Specifically, the integration of the cantilever plate structure reduced the stress in MEMS butterfly gyroscopes 346-fold, the frequency variation by 28.6%, and the bias stability approximately 2-fold [57]. Therefore, future research could further optimize the cantilever plate structure to achieve more efficient thermal stress management.

Thermal vacuum stability in space applications is also a potential research direction for MEMS gyroscopes. In harsh environments, bias drift is a significant issue. A self-compensation algorithm based on linear frequency-temperature dependence and linear amplitude–pressure dependence has been proposed, which effectively compensates for bias drift in real-time conditions. This method achieved a total bias error of 0.01°/s in the temperature range of 7–45 °C, validated through on-orbit data [152]. Future studies can explore the applicability of this self-compensation algorithm under broader temperature and pressure conditions.

Additionally, nonlinear compensation in the tiny angular velocity range is another area worth investigating. A method based on the steepest descent algorithm and Fourier series residual correction, known as the adaptive Fourier series compensation method (AFCM), significantly reduced the output nonlinearity of MEMS gyroscopes from 1150.87 to 68.89 ppm [179]. The effectiveness and superiority of this method suggest promising applications for MEMS gyroscopes in more complex dynamic environments.

Finally, reducing cross-axis sensitivity (CAS) is a critical factor for improving MEMS gyroscope performance. A design method based on the ratio matching of the drive displacement amplitude and sensing frequency difference has been proposed, which significantly reduces the CAS of a single-drive multi-axis MEMS gyroscope, from an average CAS of 0.301% to 0.045% [180]. This successful verification suggests that further optimization of design techniques could achieve even lower cross-axis sensitivity.

Future research directions for MEMS gyroscopes may include thermal stress management, thermal vacuum stability, nonlinear compensation in the small angular velocity range, and reducing cross-axis sensitivity. Exploring these areas in greater depth will contribute to enhancing the overall performance and application scope of MEMS gyroscopes.

## 6. Conclusions and Perspective

This review focuses on summarizing the research advancements in gyroscope structural forms and processing technologies from the perspective of performance metrics. The main contents of this review are as follows:

(i) An overview of the modelling principles and processes of gyroscopes based on the Coriolis force and resonance mechanisms lays a theoretical foundation for the research and development of microelectromechanical system (MEMS) gyroscopes. Moreover, the core performance indices of gyroscopes are systematically sorted, providing a standardized evaluation basis for measuring the performance of different gyroscopes and guiding their design and application.
(ii) An in-depth analysis of the evolutionary process of gyroscope designs and the distinctive features of each development stage is performed. On this basis, typical structural forms of various MEMS gyroscopes (such as single-mass-block, dual-mass-block, quadruple-mass-block, ring/disc, and hemispherical structures) are discussed, along with their corresponding processing technologies. The correlations among different structures, processing methods, and gyroscope performance indices are also clarified, revealing how structural innovation and processing progress drive performance optimization.
(iii) From the perspectives of design, fabrication, and other typical factors, analyses are provided to illustrate their impacts on performance, emphasizing the significance and challenges associated with compensation and optimization arising from internal and external influences such as design variations, process deviations, mechanical shock, thermal stress, and temperature drift.
(iv) A summary of the prominent characteristic challenges in gyroscope technologies is as follows:

(1) Characteristics: Gyroscope research presents obvious multidisciplinary properties, integrating mechanics, materials science, electronics, control theory, and other disciplines; the performance results of gyroscopes show statistical characteristics due to microscale effects and environmental interferences; internal structural defects and external environmental factors introduce errors; and corresponding error compensation methods (including hardware optimization and software algorithms) are essential to improve measurement accuracy.

(2) Challenges: Existing technical bottlenecks are analyzed in a structured way, including the difficulty of further reducing various errors (such as orthogonal errors and common-mode interference), the limitations in enhancing mechanical sensitivity, the complexity of establishing accurate system mathematical models (affected by microscale forces), and the technical difficulties in machining, levelling, and excitation of high-precision gyroscopes (such as hemispherical resonators).

Overall, the performance of gyroscopes is comprehensively determined by factors such as structural design, processing technology, assembly quality, mathematical modelling accuracy, and unpredictable environmental interferences. Although significant progress has been made in the field of gyroscope research, the inherent complexity of the technology and the continuous emergence of new application requirements mean that many issues still need to be explored and solved in the future, providing broad space for subsequent research.

## Figures and Tables

**Table 1:** Structural design impact.

Structure Type	Sensitivity (mV/°/s)	Noise Density (°/√h)	Stability Metrics	References
z-axis tuning fork MEMS gyroscope	21.76	——	ZRO: 0.002 (°/s)	[46]
Dual-Mass System	——	0.0414	Bias stability: 0.415°/h; Bandwidth: 104 Hz	[26]
Vibratory, doubly decoupled, bulk micromachined	27.6 (scale factor)	0.06	Nonlinearity: <120 ppm; Settling time < 200 ms	[8]
Doubly decoupled, silicon-glass bonded	——	0.316	Bias instability: 2°/h; ZRO drift: 0.1248°/s	[47]
Capacitive vibratory, mode-split	——	0.068	Bias instability: 0.9°/h; Phase standard deviation: 0.0004°	[48]
Tuning fork, comb-driven, silicon-on-insulator (SOI) process	23 (optimal)	——	Bias stability: 7.52 × 10^−4^°/s; Nonlinearity: 0.0062%	[49]
Vibratory, non-decoupled, dual-mode, custom metal lid	1.345	0.978	Bias instability: 9.458°/h; ZRO: 0.095°/s	[50]
Linear vibrating tuning fork MEMS gyroscope	——	——	Overshoot: −96%; Settling time: 0.036 s	[51]
Cavity optomechanical architecture	122.2	0.95	——	[52]
doubly decoupled tuning fork gyroscope	——	——	Frequency drift: 0.3 Hz/°C; Standard deviation: 0.027	[53]

**Table 2:** Control system performance.

Control Type	Performance Improvement	Stability Enhancement	Implementation Complexity	References
Self-resonant, AGC, quadrature correction	Coupling force amplitude reduced 105×; ZRO reduced	Improved precision; ZRO: 0.002 (°/s)	GA+Monte Carlo: Medium-high; Adam-LMSD: Medium	[46]
Closed-loop (no further details)	Bias stability: 2.168–0.415°/h; ARW: 0.155–0.0414°/√h	Bandwidth: 13–104 Hz; Nonlinearity: 660–59.3 ppm	Moderate	[26]
AGC-PI	Setting time < 200 ms; Amplitude fluctuation < 16 ppm	Nonlinearity < 120 ppm; Threshold: 0.005°/s	Moderate	[8]
Digital PLLs, PI (LFM vs. AM)	LFM: ZRO drift 0.1248°/s vs. AM: 10.7139°/s	LFM: Nonlinearity 329 ppm vs. AM: 1902 ppm	Moderate	[47]
MEAM (vs. CEAM), AGC+PLL	Settling time: 45.2 ms; Bias instability improved 2.4×	ARW improved 1.4×; Phase standard deviation: 0.0004°	Moderate-high	[48]
PI (GA-optimized), Adam-LMS demodulator	Sensitivity: 17.7–23 mV/(°/s); Nonlinearity: 0.0085–0.0062%	Bias stability: 0.0015–7.52 × 10^−4^°/s	High	[49]
FTR, PLL, AGC, phase delay correction	ZRO reduced by 755% to 0.095°/s	Bias instability: 9.458°/h; ARW: 0.978°/√h	Moderate-high	[50]
Adaptive PID vs. classical PID	Overshoot reduced 96%; Settling time: 0.036 s vs. 0.06 s	Similar rise time; faster stabilization	Low	[51]
Cavity optomechanical detection-based control	Sensitivity up to 122.2 mV/(°/s)	Dual-decoupled structure reduces mode coupling	Low	[52]
Incremental PID (temperature control)	Frequency drift: 0.3 Hz/°C-stable	Improved frequency stability	Combination of hardware and software, relatively complex	[53]

**Table 3:** Phase alignment effects.

Phase Error/Delay (°)	Phase Correction Mechanism	Impact on Performance Metrics	Temperature Range for Correction	References
——	Quadrature error correction	Coupling force amplitude reduced 105×; ZRO reduced	——	[46]
——	Digital PLLs; phase tracking	LFM mode: ZRO drift 0.1248°/s vs. AM: 10.7139°/s	10–50 °C	[47]
0.0004 (phase standard deviation)	Phase-locked loop	Bias instability, ARW improved; phase standard deviation: 0.0004°	——	[48]
Phase standard deviation: 0.0004°	PLL, Adam-LMS demodulator	Bias instability improved 2.4×; ARW improved 1.4×	tested under room temperature with 2 °C fluctuation	[49]
19.419 ± 0.004	Real-time PLL phase reference adjustment	ZRO reduced by 755% to 0.095°/s; ARW: 0.978°/√h; Bias instability: 9.458°/h	−20 to 70 °C	[50]

**Table 4:** Representative performance indices of MEMS gyroscopes with dual-mass, quadruple-mass, and ring/disc resonator structures.

Structure Type	Typical Performance Metrics	Advantages	Limitations	Applications	References
Dual-mass gyroscope	Scale factor: 12.5 mV/(°/s); ARW: 3°/h, 0.047°/√h, 0.18°/(h·√Hz), 0.096°/√h, 0.45°/√h, 0.021°/√h, 0.028°/√h, 0.006°/√h; Bias instability: 12°/h, 1.6°/h, 0.2°/h, 0.09°/h, 9.6°/h, 0.29°/h, <0.012°/h; Q-factor: up to 36,000; Bandwidth: 1–100 Hz; Shock resistance: >33,000 g	Vibration decoupling, high Q-factor, reduced environmental sensitivity, differential detection suppresses noise	Requires tuning, trimming, frequency matching; performance optimization still challenging	Automotive and aerospace navigation	[75,76,77,78,79,80,81,82,83,84,85,86,117]
Quadruple-mass gyroscope (QMG)	Resonant frequency: ~2 kHz; Q-factor: up to 1.2 million; ARW: 26.4°/√h, 0.72°/(h·√Hz), 0.0264°/√h, 0.28°/√h, 0.006°/√h, 0.0005°/√h; Bias instability: 0.12°/h, 5.9°/h, 0.08°/h; Measurement range: ±150°/s	Fully symmetric structure, suitable for FM detection	Requires large sensor area to ensure suspension and mode decoupling	Consumer electronics, tactical-grade sensors	[87,88,89,90,91,92,93,117]
Ring/disc resonator gyroscope (DRG)	ARW: 6°/√h, 14.4°/√h, 10.4°/(h·√Hz), 27°/√h, 3.6°/(h·√Hz), 0.015°/(h·√Hz), 0.36°/√h, 0.048°/√h, 0.138°/√h, 0.0009°/√h, 0.012°/√h, 0.083°/√h, 0.015°/√h, 0.004°/√h, 0.004°/√h, 0.026°/√h, 0.05°/√h; Bias instability: 10°/s, 0.16°/s, 20°/h, 0.01°/h, 0.08°/h, 0.11°/h, 0.015°/h, 0.87°/h, 0.85°/h, 0.42°/h; Q-factor: up to 650,000; Scale factor: 0.286 mV/(°/s), 39.8 mV/(°/s), 132 mV/(°/s), 98.1 mV/(°/s)	High precision, high Q-factor, stiffness–mass decoupling design, suitable for mass production	Complex design	High-end navigation, aerospace, UAVs	[24,94,95,96,97,98,99,100,101,102,103,104,105,106,107,108,109,110,111,112,113,114,115,116,117]

(Note: The units of Angle Random Walk (ARW) and Bias Instability (BI) are kept as reported in the original references (e.g., °/√h, °/(h·√Hz), °/h·√Hz, °/h, or °/s) to avoid misinterpretation caused by forced conversion.)

**Table 5:** Performance comparison of different MEMS gyroscope designs.

Architecture Type	Bias Stability	Angle Random Walk	Notable Performance Advantages	Notable Limitations	References
Single-mass, triaxial	——	——	Improved navigation with noise modelling	No redundancy	[141]
Mult-sensor, redundant (orthogonal/cubic/optimal)	>25% reduction	>25% reduction, 3.2×/3.7× reduction	High reliability, error reduction	Hardware complexity, missing data	[142,143,144]
Dual-mass	0.09°/h	0.0096°/√h	High stability, sensitivity	Not Multi-sensor, specialized design	[80]
9-axis	——	——	Static error (0.05°); dynamic error (0.5°)	No architecture detail	[145]

**Table 6:** Performance metrics and environmental sensitivity for different designs.

Design Type	Bias Stability	Angle Random Walk	Environmental Sensitivity	References
Triaxial inertial measurement unit	——	——	Field (auto/bike), Global navigation satellite system/inertial measurement unit	[141]
9-gyroscope, orthogonal	——	——	Simulation, fault detection and isolation test	[144]
6-gyroscope, cubic	>25% reduction	>25% reduction	Field, Global navigation satellite system, stationary/dynamic	[143]
4/5/6-gyroscope, redundant	——	Angle random walk 3.2×, rate random walk 3.7× reduction	Swing test, simulation	[142]
Dual-mass	0.09 degrees per hour	0.0096 degrees per root hour	Vacuum, 10 h, temperature/vacuum stabilized	[80]
——	——	Kalman filter output variance ≤ 30%	——	[147]
9-axis	——	——	static/dynamic, orientation	[145]

**Table 7:** Silicon-based MEMS processes.

Process Type	Key Parameters	Quality Metrics	Limitations	References
SOG: patterning, anodic bonding, DRIE, CMP	Silicon thickness: 50 μm, roughness: 1.13 nm	Q~12,000, frequency~4 kHz	Lag effect, notching, etch endpoint control	[146]
SOI, single crystal silicon, digital control	——	Q~100,000, bias stability 0.18°/h	——	[156]

**Table 8:** Glass-based resonator fabrication.

Process Type	Key Parameters	Quality Metrics	Limitations	References
Laser-induced etching (LIE) on fused silica	Etch width 11 μm, temperature 20–70 °C, 10–30 min	Q > 810,000, overload > 15,000 g, 45 MPa	Subsurface cracks, etch control, vacuum requirement	[157]
Precision machining, polishing, etching	Roundness 0.17 μm, roughness 15.2 nm	Q = 3.11 × 10^7^	Surface loss, stress, geometry limits	[158]
Fused silica, hemispherical resonator	Shell radius 10–15 mm, damage layer 0–100 μm	QTED reduction up to 92.5%	Wall thickness nonuniformity	Geometric parameters

**Table 9:** Advanced bonding techniques.

Process Type	Key Parameters	Quality Metrics	Limitations	References
Anodic bonding (silicon to Pyrex)	Bonding after wet etch, pre-DRIE	Well-defined structures, no footing	Wafer misalignment, lapping damage	[146]
Direct bonding (fused silica)	Resonator to substrate	High process precision	Bonding error, stress	[157]

**Table 10:** Surface quality effects.

Performance Metric	Process Influence	Achieved Results	Limiting Factors	References
Surface roughness	Chemical Mechanical Polishing (CMP), cerium oxide polish	1.13 nm	Lapping damage, misalignment	[146]
Subsurface cracks, etch width	Laser-Induced Etching (LIE), ultrasonic, annealing	Etch width 11 μm, crack width 4 μm	Subsurface cracks, etch control	[157]
Surface roughness	Precision polishing/etching	15.2 nm	Surface loss, stress	[158]

**Table 11:** Structural integrity.

Performance Metric	Process Influence	Achieved Results	Limiting Factors	References
Overload, strength	Laser-induced etching (LIE), structure compensation	>15,000 g, 45 MPa	Crack formation, etch uniformity	[157]
Mechanical performance	DRIE optimization	No notching /footing	Lag effect, endpoint control	[146]

**Table 12:** Q-factor optimization.

Performance Metric	Process Influence	Achieved Results	Limiting Factors	References
Q-factor	DRIE/CMP optimization	10,437–12,058	Lag effect, etch uniformity	[146]
Q-factor	Laser-induced etching (LIE), structure compensation	817,000–819,000	Subsurface cracks, vacuum	[157]
Q-factor	Precision machining /polishing	3.11 × 10^7^	Surface loss, stress, geometry	[158]
Q-factor	SOI, digital compensation	~100,000	——	[156]
Q-factor stability	In situ Joule tuning	ΔQ~150 ppm	Not fabrication- limited	[159]

## Data Availability

No new data were created in this review article.

## References

[1] Söderkvist J. (1994). Micromachined gyroscopes. Sens. Actuators A Phys..

[2] Lawrence A. (1993). Modern Inertial Technology: Navigation, Guidance, and Control.

[3] Pansiot J., Zhang Z.Q., Lo B., Yang G. (2011). WISDOM: Wheelchair inertial sensors for displacement and orientation monitoring. Meas. Sci. Technol..

[4] Xu Y., Chen X.Y., Wang Y.M. (2016). Two-mode navigation method for low-cost inertial measurement unit-based indoor pedestrian navigation. Simulation.

[5] Fan L.S., Tai Y.C., Muller R.S. (1988). Integrated movable micromechanical structures for sensors and actuators. IEEE Trans. Electron Devices.

[6] Tatar E., Alper S.E., Akin T. (2012). Quadrature-error compensation and corresponding effects on the performance of fully decoupled MEMS gyroscopes. J. Microelectromech. Syst..

[7] Saukoski M., Aaltonen L., Halonen K.A.I. (2008). Effects of synchronous demodulation in vibratory MEMS gyroscopes: A theoretical study. IEEE Sens. J..

[8] Cui J., Chi X.Z., Ding H.T., Lin L.T., Yang Z.C., Yan G.Z. (2009). Transient response and stability of the AGC-PI closed-loop controlled MEMS vibratory gyroscopes. J. Micromech. Microeng..

[9] Hou Z.Q., Xiao D.B., Wu X.Z., Dong P.T., Chen Z.H., Niu Z.Y., Zhang X. (2011). Effect of axial force on the performance of micromachined vibratory rate gyroscopes. Sensors.

[10] Xiao D.B., Su J.B., Chen Z.H., Hou Z.Q., Wang X.H., Wu X.Z. (2013). Improvement of mechanical performance for vibratory microgyroscope based on sense mode closed-loop control. J. Micro/Nanolith. MEMS MOEMS.

[11] Feng R., Qiu A.P., Shi Q., Su Y. (2011). A theoretical and experimental study on temperature dependent characteristics of silicon MEMS gyroscope drive mode. Adv. Mater. Res..

[12] Bernstein J., Cho S., King A.T., Kourepenis A., Maciel P., Weinberg M. (1993). A micromachined comb-drive tuning fork rate gyroscope. Proceedings of the IEEE Micro Electro Mechanical Systems.

[13] Johnson B.R., Cabuz E., French H.B., Supino R. (2010). Development of a MEMS gyroscope for northfinding applications. Proceedings of the IEEE/ION Position, Location and Navigation Symposium.

[14] Geiger W., Folkmer B., Sobe U., Sandmaier H., Lang W. (1998). New designs of micromachined vibrating rate gyroscopes with decoupled oscillation modes. Sens. Actuators A Phys..

[15] Antonello R., Oboe R., Prandi L., Biganzoli F. (2009). Automatic Mode Matching in MEMS Vibrating Gyroscopes Using Extremum-Seeking Control. IEEE Trans. Ind. Electron..

[16] Riaz K., Bazaz S.A., Saleem M.M., Shakoor R.I. (2011). Design, damping estimation and experimental characterization of decoupled 3-DoF robust MEMS gyroscope. Sens. Actuators A Phys..

[17] Shen Q., Li H., Hao Y.C., Yuan W.Z., Chang H.L. (2016). Bias contribution modeling for a symmetrical micromachined Coriolis vibratory gyroscope. IEEE Sens. J..

[18] Cetin H., Yaralioglu G.G. (2017). Analysis of vibratory gyroscopes: Drive and sense mode resonance shift by coriolis force. IEEE Sens. J..

[19] Tu Y.H., Peng C.C. (2021). An ARMA-based digital twin for MEMS gyroscope drift dynamics modeling and real-time compensation. IEEE Sens. J..

[20] Georgy J., Noureldin A., Korenberg M.J., Bayoumi M.M. (2010). Modeling the stochastic drift of a MEMS-based gyroscope in gyro/odometer/GPS integrated navigation. IEEE Trans. Intell. Transp. Syst..

[21] Li X., Li Z. (2014). Vector-aided in-field calibration method for low-end MEMS gyros in attitude and heading reference systems. IEEE Trans. Instrum. Meas..

[22] Shkel A.M. (2006). Type I and type II micromachined vibratory gyroscopes. Proceedings of the 2006 IEEE/ION Position, Location, And Navigation Symposium.

[23] Wang S.H., Al Farisi M.S., Tsukamoto T., Tanaka S. (2020). Roll/pitch rate integrating mems gyroscope using dynamically balanced dual-mass resonator. Proceedings of the 2020 IEEE International Symposium on Inertial Sensors and Systems (INERTIAL).

[24] Fan B., Guo S.W., Cheng M.M., Yu L., Zhou M., Hu W.Y., Chen Z.A., Xu D.C. (2019). A novel high-symmetry cobweb-like disk resonator gyroscope. IEEE Sens. J..

[25] Yang J., Hamelin B., Ayazi F. (2020). Investigating elastic anisotropy of 4H-SiC Using Ultra-High Q bulk acoustic wave resonators. J. Microelectromech. Syst..

[26] Cao H.L., Xue R.H., Cai Q., Gao J.Y., Zhao R., Shi Y.B., Huang K., Shao X.L., Shen C. (2020). Design and experiment for dual-mass MEMS gyroscope sensing closed-loop system. IEEE Access.

[27] Shao X.L., Shi Y., Zhang W.D., Cao H.L. (2021). Neurodynamic approximation-based quantized control with improved transient performances for microelectromechanical system gyroscopes: Theory and experimental results. IEEE Trans. Ind. Electron..

[28] Sung S., Sung W.T., Kim C., Yun S., Lee Y.J. (2009). On the mode-matched control of MEMS vibratory gyroscope via phase-domain analysis and design. IEEE/ASME Trans. Mechatron..

[29] Trusov A.A., Schofield A.R., Shkel A.M. (2011). Micromachined rate gyroscope architecture with ultra-high quality factor and improved mode ordering. Sens. Actuators A Phys..

[30] Yoon S., Lee S., Perkins N., Najafi K. (2010). Analysis and wafer-level design of a high-order silicon vibration isolator for resonating MEMS devices. J. Micromech. Microeng..

[31] Xie H.K., Fedder G.K. (2003). Fabrication, characterization, and analysis of a DRIE CMOS-MEMS gyroscope. IEEE Sens. J..

[32] Sharma A., Zaman M.F., Ayazi F. (2009). A sub-0.2°/hr bias drift micromechanical silicon gyroscope with automatic CMOS mode-matching. IEEE J. Solid-State Circuits.

[33] Sharma M., Sarraf E.H., Baskaran R., Cretu E. (2012). Parametric resonance: Amplification and damping in MEMS gyroscopes. Sens. Actuators A Phys..

[34] Dion F., Martel S., Denatale J. (2018). 200mm High performance inertial sensor manufacturing process. Proceedings of the 2018 IEEE International Symposium on Inertial Sensors and Systems (INERTIAL).

[35] Denatale J., Martel S., Dion F., Lachance J. (2020). Manufacturing transition of high-performance MEMS gyroscopes. Proceedings of the 2020 IEEE/ION Position, Location and Navigation Symposium (PLANS).

[36] Asadian M.H., Wang Y.S., Shkel A.M. (2019). Development of 3D fused quartz hemi-toroidal shells for high-Q resonators and gyroscopes. J. Microelectromech. Syst..

[37] Chen J.L., Tsukamoto T., Tanaka S. (2019). Quad mass gyroscope with 16 ppm frequency mismatch trimmed by focus ion beam. Proceedings of the 2019 IEEE International Symposium on Inertial Sensors and Systems (INERTIAL).

[38] Feng H.Z., Lou W.Z., Wang D.K., Zheng F.Q., Liao M.H. (2018). System reliability analysis of MEMS gyroscope with multiple failure modes. Proceedings of the 2018 10th International Conference on Modelling, Identification and Control (ICMIC).

[39] Li J., Broas M., Makkonen J., Mattila T.T., Hokka J., Paulasto-Kröckel M. (2014). Shock impact reliability and failure analysis of a three-axis MEMS gyroscope. J. Microelectromech. Syst..

[40] Patel C., Mccluskey P., Lemus D. (2010). Performance and reliability of mems gyroscopes at high temperatures. Proceedings of the 2010 12th IEEE Intersociety Conference on Thermal and Thermomechanical Phenomena in Electronic Systems.

[41] Betta G., Capriglione D., Carratù M., Catelani M., Ciani L., Patrizi G., Pietrosanto A., Sommella P. (2022). Stress testing for performance analysis of orientation estimation algorithms. IEEE Trans. Instrum. Meas..

[42] Cao H.L. (2023). Dual-Mass Linear Vibration Silicon Based MEMS Gyroscope.

[43] Cao H.L., Li H.S. (2013). Investigation of a vacuum packaged MEMS gyroscope architecture’s temperature robustness. Int. J. Appl. Electromagn. Mech..

[44] Yang B., Wang S.R., Li H.S., Zhou B.L. (2009). Mechanical-thermal noise in drive-mode of a silicon micro-gyroscope. Sensors.

[45] Yazdi N., Ayazi F., Najafi K. (1998). Micromachined inertial sensors. Proc. IEEE.

[46] Gu H.Y., Su W., Zhao B.L., Zhou H., Liu X.X. (2020). A design methodology of digital control system for MEMS gyroscope based on multi-objective parameter optimization. Micromachines.

[47] Cao H.L., Li H., Wang S.R., Yang B., Huang L.B. (2013). Structure model and system simulation of MEMS gyroscope. J. Chin. Inert. Technol..

[48] Wang X.T., Zheng X.D., Shen Y.J., Xia C.H., Liu G.W., Jin Z.H., Ma Z.P. (2022). A digital control structure for Lissajous frequency-modulated mode MEMS gyroscope. IEEE Sens. J..

[49] Ma W., Lin Y.Y., Liu S.Q., Zheng X.D., Jin Z.H. (2016). A novel oscillation control for MEMS vibratory gyroscopes using a modified electromechanical amplitude modulation technique. J. Micromech. Microeng..

[50] Xu P.F., Wei Z.Y., Guo Z.Y., Jia L., Han G.W., Si C.W., Ning J., Yang F.H. (2021). A real-time circuit phase delay correction system for MEMS vibratory gyroscopes. Micromachines.

[51] Yang K., Li J.H., Yang J.J., Xu L.X. (2024). Research on adaptive closed-loop control of microelectromechanical system gyroscopes under temperature disturbance. Micromachines.

[52] Yan X., Huang W.Y., Li Z., Chen K., Deng G.W., Wen G.J., Huang Y.J. (2023). Novel high-precision micro-gyroscope based on cavity optomechanical system. Sci. Sin. Phys. Mech. Astron..

[53] Rui G., He C.H., Liu D.C., Zhao Q.C., Yang Z.C., Yan G.Z. (2015). A temperature control system used for improving resonant frequency drift of MEMS gyroscopes. Proceedings of the 10th IEEE International Conference on Nano/Micro Engineered and Molecular Systems.

[54] Naumenko D., Tkachenko A., Lysenko I., Kovalev A. (2023). Development and research of the sensitive element of the MEMS gyroscope manufactured using SOI technology. Micromachines.

[55] Kim C., Park J., Kim T., Kim J.S., Seong J., Shim H., Ko H., Cho D.I. (2022). Development and evaluation of haltere-mimicking gyroscope for three-axis angular velocity sensing using a haltere-mimicking structure pair. Bioinspir. Biomim..

[56] Gao Y., Huang L.B., Ding X.K., Li H.S. (2018). Design and implementation of a dual-mass MEMS gyroscope with high shock resistance. Sensors.

[57] Kuang Y.B., Huo X.Y., Guo W.T., Li X.X., He J.Y., Mao Q., Ma X.L., Liu J. (2025). Research on the method of optimizing the stress and improving the performance for MEMS gyroscope based on the cantilever-plate structure. Micromachines.

[58] Greiff P., Boxenhorn B., King T., Niles L. (1991). Silicon monolithic micromechanical gyroscope. Proceedings of the TRANSDUCERS ‘91: 1991 International Conference on Solid-State Sensors and Actuators. Digest of Technical Papers.

[59] Tanaka K., Mochida Y., Sugimoto S., Moriya K., Hasegawa T., Atsuchi K., Ohwada K. (1995). A micromachined vibrating gyroscope. Proceedings of the IEEE Micro Electro Mechanical Systems.

[60] Mochida Y., Tamura M., Ohwada K. (1999). A micromachined vibrating rate gyroscope with independent beams for the drive and detection modes. Proceedings of the Technical Digest. IEEE International MEMS 99 Conference. Twelfth IEEE International Conference on Micro Electro Mechanical Systems (Cat. No.99CH36291).

[61] Geiger W., Butt W.U., Gaißer A., Frech J., Braxmaier M., Link T., Kohne A., Nommensen P., Sandmaier H., Lang W. (2002). Decoupled microgyros and the design principle DAVED. Sens. Actuators A Phys..

[62] Alper S.E., Akin T. (2002). A symmetric surface micromachined gyroscope with decoupled oscillation modes. Sens. Actuators A Phys..

[63] Alper S.E., Silay K.M., Akin T. (2006). A low-cost rate-grade nickel microgyroscope. Sens. Actuators A Phys..

[64] Xiong B., Che L.F., Wang Y.L. (2003). A novel bulk micromachined gyroscope with slots structure working at atmosphere. Sens. Actuators A Phys..

[65] Sung W.T., Sung S., Lee J.G., Kang T. (2007). Design and performance test of a MEMS vibratory gyroscope with a novel AGC force rebalance control. J. Micromech. Microeng..

[66] Alper S.E., Temiz Y., Akin T. (2008). A compact angular rate sensor system using a fully decoupled silicon-on-glass MEMS gyroscope. J. Microelectromech. Syst..

[67] Cui J., Guo Z.Y., Zhao Q.C., Yang Z.C., Hao Y.L., Yan G.Z. (2011). Force rebalance controller synthesis for a micromachined vibratory gyroscope based on sensitivity margin specifications. J. Microelectromech. Syst..

[68] He C.H., Zhao Q.C., Liu Y.X., Yang Z.C., Yan G.Z. (2013). Closed loop control design for the sense mode of micromachined vibratory gyroscopes. Sci. China Technol. Sci..

[69] Li Z.H., Yang Z.C., Xiao Z.X., Hao Y.L., Li T., Wu G.Y., Wang Y.Y. (2000). A bulk micromachined vibratory lateral gyroscope fabricated with wafer bonding and deep trench etching. Sens. Actuators A Phys..

[70] Liu X.S., Yang Z.C., Chi X.Z., Cui J., Ding H.T., Guo Z.Y., Lv B., Lin L.T., Zhao Q.C., Yan G.Z. (2009). A doubly decoupled lateral axis micromachined gyroscope. Sens. Actuators A Phys..

[71] Xie J.B., Shen Q., Hao Y.C., Chang H.L., Yuan W.Z. (2015). Design, fabrication and characterization of a low-noise Z-axis micromachined gyroscope. Microsyst. Technol..

[72] Li X.X., Bao M.H., Yang H., Shen S.Q., Lu D.R. (1999). A micromachined piezoresistive angular rate sensor with a composite beam structure. Sens. Actuators A Phys..

[73] Yang H., Bao M.H., Yin H., Shen S.Q. (2002). A novel bulk micromachined gyroscope based on a rectangular beam-mass structure. Sens. Actuators A Phys..

[74] Acar C., Shkel A. (2009). MEMS Vibratory Gyroscopes: Structural Approaches to Improve Robustness.

[75] Geen J.A., Sherman S.J., Chang J.F., Lewis S.R. (2002). Single-chip surface micromachined integrated gyroscope with 50/spl deg//h Allan deviation. IEEE J. Solid-State Circuits.

[76] Zhang H., Yin L., Chen W.P., Fu Q., Zhang W.B. (2024). Monolithic integrated interface ASIC with quadrature error correction for MEMS dual-mass vibration gyroscope. IEEE Sens. J..

[77] Hanse J.G. (2004). Honeywell MEMS inertial technology & product status. Proceedings of the PLANS 2004. Position Location and Navigation Symposium (IEEE Cat. No. 04CH37556).

[78] Zaman M.F., Sharma A., Ayazi F. (2006). High performance matched-mode tuning fork gyroscope. Proceedings of the 19th IEEE International Conference on Micro Electro Mechanical Systems.

[79] Sharma A., Zaman M.F., Ayazi F. (2007). A 0.2/hr micro-gyroscope with automatic CMOS mode matching. Proceedings of the 2007 IEEE International Solid-State Circuits Conference. Digest of Technical Papers.

[80] Wang D.M., Efimovskaya A., Shkel A.M. (2019). Amplitude amplified dual-mass gyroscope: Design architecture and noise mitigation strategies. Proceedings of the 2019 IEEE International Symposium on Inertial Sensors and Systems (INERTIAL).

[81] Wu G.Q., Chua G.L., Gu Y.D. (2017). A dual-mass fully decoupled MEMS gyroscope with wide bandwidth and high linearity. Sens. Actuators A Phys..

[82] Koumela A., Poulain C., Le Goc C., Verdot T., Joet L., Rey P., Berthelot A., Jourdan G. (2016). Resilience to vibration of a tuning fork MEMS gyroscope. Procedia Eng..

[83] Efimovskaya A., Wang D.M., Shkel A.M. (2020). Mechanical trimming with focused ion beam for permanent tuning of MEMS dual-mass gyroscope. Sens. Actuators A Phys..

[84] Chen J.L., Tsukamoto T., Tanaka S. (2022). Triple mass resonator for electrostatic quality factor tuning. J. Microelectromech. Syst..

[85] Wang S.H., Chen J.L., Tsukamoto T., Langfelder G., Tanaka S. (2023). Challenges in implementing pitch/roll rate integrating gyroscopes: A case study on a new dynamically balanced dual-mass resonator. IEEE Sens. J..

[86] Vercier N., Chaumet B., Leverrier B., Bouyat S. (2020). A new silicon axisymmetric gyroscope for aerospace applications. Proceedings of the 2020 DGON Inertial Sensors and Systems (ISS).

[87] Prikhodko I.P., Zotov S.A., Trusov A.A., Shkel A.M. (2012). Foucault pendulum on a chip: Rate integrating silicon MEMS gyroscope. Sens. Actuators A Phys..

[88] Cho J.Y. (2012). High-Performance Micromachined Vibratory Rate- and Rate-Integrating Gyroscopes. Ph.D. Thesis.

[89] Zhou B., Zhang T., Yin P., Chen Z.Y., Song M.L., Zhang R. (2016). Innovation of flat gyro: Center support quadruple mass gyroscope. Proceedings of the 2016 IEEE International Symposium on Inertial Sensors and Systems.

[90] Taheri-Tehrani P., Kline M., Izyumin I., Eminoglu B., Yeh Y.C., Yang Y.S., Chen Y.H., Flader I., Ng E.J., Kenny T.W. (2016). Epitaxially-encapsulated quad mass gyroscope with nonlinearity compensation. Proceedings of the 2016 IEEE 29th International Conference on Micro Electro Mechanical Systems (MEMS).

[91] Wu G.Q., Chua G.L., Singh N., Gu Y.D. (2018). A quadruple mass vibrating MEMS gyroscope with symmetric design. IEEE Sens. Lett..

[92] Gianollo M., Mastri V., Zega V., Bestetti M., Falorni L., Langfelder G. (2021). Miniaturized quadruple mass gyroscopes: Challenges and implementation. Proceedings of the 2021 IEEE Sensors.

[93] Knight R.R., Rudy R.Q., Pulskamp J.S., Benoit R.R., Devoe D.L., Lau E. (2024). Quadruple mass gyroscope angle random walk reduction through linearized transduction. J. Microelectromech. Syst..

[94] Putty M.W. (1995). A Micromachined Vibrating Ring Gyroscope. Ph.D. Thesis.

[95] Juneau T., Pisano A.P., Smith J.H. (1997). Dual axis operation of a micromachined rate gyroscope. Proceedings of the International Solid State Sensors and Actuators Conference (Transducers ‘97).

[96] He G.H., Najafi K. (2002). A single-crystal silicon vibrating ring gyroscope. Proceedings of the Technical Digest. MEMS 2002 IEEE International Conference. Fifteenth IEEE International Conference on Micro Electro Mechanical Systems (Cat. No.02CH37266).

[97] Zhao Q.C., Liu X.S., Lin L.T., Guo Z.Y., Cui J., Chi X.Z., Yang Z.C., Yan G.Z. (2009). A doubly decoupled micromachined vibrating wheel gyroscope. Proceedings of the TRANSDUCERS 2009—2009 International Solid-State Sensors, Actuators and Microsystems Conference.

[98] Cho J., Gregory J.A., Najafi K. (2011). Single-crystal-silicon vibratory cylinderical rate integrating gyroscope (CING). Proceedings of the 2011 16th International Solid-State Sensors, Actuators and Microsystems Conference.

[99] Han F.T., Liu Y.F., Wang L., Ma G.Y. (2012). Micromachined electrostatically suspended gyroscope with a spinning ring-shaped rotor. J. Micromech. Microeng..

[100] Nitzan S., Ahn C.H., Su T.H., Li M., Ng E.J., Wang S., Yang Z.M., O’brien G., Boser B.E., Kenny T.W. (2013). Epitaxially-encapsulated polysilicon disk resonator gyroscope. Proceedings of the 2013 IEEE 26th International Conference on Micro Electro Mechanical Systems (MEMS).

[101] Su T.H., Nitzan S.H., Taheri-Tehrani P., Kline M.H., Boser B.E., Horsley D.A. (2014). Silicon MEMS disk resonator gyroscope with an integrated CMOS analog front-end. IEEE Sens. J..

[102] Challoner A.D., Ge H.H., Liu J.Y. (2014). Boeing disc resonator gyroscope. Proceedings of the 2014 IEEE/ION Position, Location and Navigation Symposium—PLANS 2014.

[103] Zhou X., Xiao D.B., Wu X.Z., Wu Y.L., Hou Z.Q., He K.X., Li Q.S. (2016). Stiffness-mass decoupled silicon disk resonator for high resolution gyroscopic application with long decay time constant (8.695s). Appl. Phys. Lett..

[104] Zhou X., Xiao D.B., Li Q.S., Hou Z.Q., He K.X., Chen Z.H., Wu Y.L., Wu X.Z. (2018). Decaying time constant enhanced MEMS disk resonator for high precision gyroscopic application. IEEE/ASME Trans. Mechatron..

[105] Li Q.S., Xiao D.B., Zhou X., Ou F., Hou Z.Q., Wu X.Z. (2017). A novel honeycomb-like disk resonant gyroscope. Proceedings of the 2017 19th International Conference on Solid-State Sensors, Actuators and Microsystems (TRANSDUCERS).

[106] Xu Y., Li Q.S., Zhou X., Gao K., Wang P., Zhang Y.M., Hou Z.Q., Wu X.Z., Xiao D.B. (2019). Stiffness-mass decoupled honeycomb-like disk resonator gyroscope. Proceedings of the 2019 IEEE 32nd International Conference on Micro Electro Mechanical Systems (MEMS).

[107] Xu Y., Li Q.S., Wang P., Zhang Y.M., Zhou X., Yu L., Wu X.Z., Xiao D.B. (2021). 0.015 degree-per-hour honeycomb disk resonator gyroscope. IEEE Sens. J..

[108] Kaji S., Gando R., Masunishi K., Ogawa E., Miyazaki F., Hiraga H., Tomizawa Y., Shibata H. (2020). A <100 PPB/K frequency-matching temperature stability MEMS rate integrating gyroscope enabled by donut-mass structure. Proceedings of the 2020 IEEE 33rd International Conference on Micro Electro Mechanical Systems (MEMS).

[109] Lin D., Macdonald R., Calbaza D., Scherer B., Johnson T., Toepfer T., Shaddock D., Andarawis E. (2020). Sub-Degree-per-hour mems gyroscope for measurement while drilling at 300 °C. Proceedings of the 2020 IEEE/ION Position, Location and Navigation Symposium (PLANS).

[110] Wang J.B., Chen L., Zhang M., Chen D.Y. (2010). A micro-machined vibrating ring gyroscope with highly symmetric structure for harsh environment. Proceedings of the 2010 IEEE 5th International Conference on Nano/Micro Engineered and Molecular Systems.

[111] Liu J.L., Chen D.Y., Wang J.B. (2012). Regulating parameters of electromagnetic micromachined vibrating ring gyroscope by feedback control. Micro Nano Lett..

[112] Wang H., Quan H.Y., Zhou J.Q., Zhang L., Xie J.B., Chang H.L. (2022). A wafer-level vacuum packaged MEMS disk resonator gyroscope with 0.42°/h bias instability within±300°/s full scale. IEEE Trans. Ind. Electron..

[113] Wen H.R., Daruwalla A., Liu C.S., Ayazi F. (2020). A hermetically-sealed 2.9MHz N = 3 disk BAW gyroscope with sub-degree-per-hour bias instability. Proceedings of the 2020 IEEE 33rd International Conference on Micro Electro Mechanical Systems (MEMS).

[114] Ren X.J., Zhou X., Tao Y., Li Q.S., Wu X.Z., Xiao D.B. (2021). Radially pleated disk resonator for gyroscopic application. J. Microelectromech. Syst..

[115] Gu L.T., Zhang W.P., Lu H.L., Wu Y.T., Fan C.Y. (2022). Flower-like disk resonator for gyroscopic application. Rev. Sci. Instrum..

[116] Obitani K., Araya K., Yachi M., Tsuchiya T. (2021). Piezoelectric disk gyroscope fabricated with single-crystal lithium niobate. J. Microelectromech. Syst..

[117] Wei W.Q., Tian H.M., Chen K., Wang F.E., Lu Z.H., Chen F., Cao H.L., Xie H.K. (2025). Triaxial MEMS gyroscopes: A review. IEEE Sens. J..

[118] Meyer A.D., Rozelle D.M. Milli-HRG inertial navigation system. Proceedings of the 2012 IEEE/ION Position, Location and Navi-gation Symposium.

[119] Rozelle D.M. (2009). The hemispherical resonator gyro: From wineglass to the planets. Spaceflight Mech..

[120] Li W., Xi X., Lu K., Shi Y., Hou Z.Q., Wu Y.L., Wu X.Z., Xiao D.B. (2019). A novel high transduction efficiency micro shell resonator gyroscope with 16 t-shape masses using out-of-plane electrodes. IEEE Sens. J..

[121] Cho J.Y., Singh S., Woo J.K., He G.H., Najafi K. (2020). 0.00016 deg/√hr angle random walk (ARW) and 0.0014 deg/hr bias instability (BI) from a 5.2M-Q and 1-cm precision shell integrating (PSI) gyroscope. Proceedings of the 2020 IEEE International Symposium on Inertial Sensors and Systems (INERTIAL).

[122] Meyer A.D., Rozelle D.M., Trusov A.A., Sakaida D.K. (2018). milli-HRG inertial sensor assembly—A reality. Proceedings of the 2018 IEEE/ION Position, Location and Navigation Symposium (PLANS).

[123] Foloppe Y., Lenoir Y. (2019). HRG Crystal™ DUAL CORE: Rebooting the INS revolution. Proceedings of the 2019 DGON Inertial Sensors and Systems (ISS).

[124] Senkal D. (2015). Micro-Glassblowing Paradigm for Realization of Rate Integrating Gyroscopes. Ph.D. Thesis.

[125] Shao P., Tavassoli V., Mayberry C.L., Ayazi F. (2015). A 3D-HARPSS polysilicon microhemispherical shell resonating gyroscope: Design, fabrication, and characterization. IEEE Sens. J..

[126] Liu Z.Y., Zhang W.P., Cui F., Tang J. (2020). Three-dimensional micromachined diamond birdbath shell resonator on silicon substrate. Microsyst. Technol..

[127] Gray J.M., Houlton J.P., Gertsch J.C., Brown J.J., Rogers C.T., George S.M., Bright V.M. (2014). Hemispherical micro-resonators from atomic layer deposition. J. Micromech. Microeng..

[128] Tavassoli V., Hamelin B., Ayazi F. (2016). Substrate-decoupled 3D micro-shell resonators. Proceedings of the 2016 IEEE SENSORS.

[129] Hassan J.N.A., Huang W.Y., Wang M.Y., Zhang S.Y., Wen G.J., Huang Y.J. (2024). Optomechanical gyroscope based on micro-hemispherical shell and optical ring resonators. IEEE Photonics J..

[130] Eklund E.J., Shkel A.M. (2007). Glass blowing on a wafer level. J. Microelectromech. Syst..

[131] Cho J.Y., Yan J.L., Gregory J.A., Eberhart H.W., Peterson R.L., Najafi K. (2013). 3-dimensional blow torch-molding of fused silica microstructures. J. Microelectromech. Syst..

[132] Cho J.Y., Najafi K. (2015). A high-q all-fused silica solid-stem wineglass hemispherical resonator formed using micro blow torching and welding. Proceedings of the 2015 28th IEEE International Conference on Micro Electro Mechanical Systems (MEMS).

[133] Nagourney T., Cho J.Y., Shiari B., Darvishian A., Najafi K. (2017). 259 Second ring-down time and 4.45 million quality factor in 5.5 kHz fused silica birdbath shell resonator. Proceedings of the 2017 19th International Conference on Solid-State Sensors, Actuators and Microsystems (TRANSDUCERS).

[134] Shi Y., Xi X., Li B., Chen Y.M., Wu Y.L., Xiao D.B., Wu X.Z., Lu K. (2021). Micro hemispherical resonator gyroscope with teeth-like tines. IEEE Sens. J..

[135] Cho J.Y., Woo J.K., He G.H., Yang D., Boyd C., Singh S., Darvishian A., Shiari B., Najafi K. (2019). 1.5-million Q-factor vacuum-packaged birdbath resonator gyroscope (BRG). Proceedings of the 2019 IEEE 32nd International Conference on Micro Electro Mechanical Systems (MEMS).

[136] Luo B., Shang J.T., Zhang Y.Z. (2015). Hemispherical glass shell resonators fabricated using chemical foaming process. Proceedings of the 2015 IEEE 65th Electronic Components and Technology Conference (ECTC).

[137] Crescenzi R., Castellito G.V., Quaranta S., Balucani M. (2020). Design of a tri-axial surface micromachined MEMS vibrating gyroscope. Sensors.

[138] Hu H.T., Calusi B., Bagolini A., Pantano M.F. (2025). Design, analysis, and simulation of a MEMS tuning fork gyroscope with a mechanical amplification structure. Micromachines.

[139] Xu P.F., Si C.W., He Y.R., Wei Z.Y., Jia L., Han G.W., Ning J., Yang F.H. (2021). A novel high-Q dual-mass MEMS tuning fork gyroscope based on 3D wafer-level packaging. Sensors.

[140] Tian H.M., Zhang Z.H., Liu L., Wei W.Q., Cao H.L. (2024). Design and implementation of a CMOS-MEMS out-of-plane detection gyroscope. Micromachines.

[141] Suvorkin V., Garcia-Fernandez M., González-Casado G., Li M.W., Rovira-Garcia A. (2024). Assessment of noise of MEMS IMU sensors of different grades for GNSS/IMU navigation. Sensors.

[142] Xue L., Yang B., Wang X.G., Shan B., Gao J.A., Chang H.L., Yao Y.F. (2023). Design of optimal estimation algorithm for multi-sensor fusion of a redundant MEMS gyro system. IEEE Sens. J..

[143] De Alteriis G., Accardo D., Conte C., Schiano Lo Moriello R. (2021). Performance enhancement of consumer-grade MEMS sensors through geometrical redundancy. Sensors.

[144] Cheng J.H., Dong J.L., Landry R.J., Chen D.D. (2014). A novel optimal configuration form redundant MEMS inertial sensors based on the orthogonal rotation method. Sensors.

[145] Lin Z.R., Xiong Y.S., Dai H.D., Xia X.K. (2017). An experimental performance evaluation of the orientation accuracy of four nine-axis MEMS motion sensors. Proceedings of the 2017 5th International Conference on Enterprise Systems (ES).

[146] Ma Z.B., Wang Y.N., Shen Q., Zhang H., Guo X.T. (2018). Key processes of silicon-on-glass MEMS fabrication technology for gyroscope application. Sensors.

[147] Diao Z.L., Quan H.Y., Lan L.D., Han Y.F. (2013). Analysis and compensation of MEMS gyroscope drift. Proceedings of the 2013 Seventh International Conference on Sensing Technology (ICST).

[148] Saqib M., Mubasher Saleem M., Mazhar N., Awan S.U., Shahbaz Khan U. (2018). Design and analysis of a high-gain and robust multi-DOF electro-thermally actuated MEMS gyroscope. Micromachines.

[149] Xu Y.Y., Lin J., He C.H., Wu H., Huang Q.W., Yan G.Z. (2024). Design of a shock-protected structure for MEMS gyroscopes over a full temperature range. Micromachines.

[150] Wang L., Pan Y.H., Li K., He L.L., Wang Q.Y., Wang W.D. (2024). Modeling and reliability analysis of MEMS gyroscope rotor parameters under vibrational stress. Micromachines.

[151] Chen L.Q., Miao T.Q., Li Q.S., Wang P., Wu X.Z., Xi X., Xiao D.B. (2022). A temperature drift suppression method of mode-matched MEMS gyroscope based on a combination of mode reversal and multiple regression. Micromachines.

[152] Liu J.L., Fu M.R., Meng C., Li J.P., Li K., Hu J., Chen X.J. (2020). Consideration of thermo-vacuum stability of a MEMS gyroscope for space applications. Sensors.

[153] Chen C., Zhou J.Q., Wang H.Y., Fan Y.Y., Song X.Y., Xie J.B., Bäck T., Wang H. (2024). Machine learning-driven discovery of high-performance MEMS disk resonator gyroscope structural topologies. Microsyst. Nanoeng..

[154] Chang H.L., Xue L., Qin W., Yuan G.M., Yuan W.Z. (2008). An integrated MEMS gyroscope array with higher accuracy output. Sensors.

[155] Huang F.R., Wang Z., Xing L.R., Gao C. (2022). A MEMS IMU Gyroscope Calibration Method Based on Deep Learning. IEEE Trans. Instrum. Meas..

[156] Fan Q., Lin C., Liu M.X., Su Y., Zhao W.L., Zheng D.W. (2018). High performance MEMS disk gyroscope with force-to-rebalance operation mode. Proceedings of the 2018 IEEE SENSORS.

[157] Wang C.X., Wu K., Wang X.Y., Li Q.S., Zhang Y.M., Wu Y.L., Wu X.Z., Xiao D.B. (2024). A MEMS disk gyroscope with high fabrication precision, high quality factor (>810k) and high overload characteristic (>15000 g). J. Phys. Conf. Ser..

[158] Liu J.H., Qu T.L., Xiong C.X. (2023). Precision machining technology of high quality factor hemispherical resonator. Proceedings of the 2nd International Forum of Young Scientists on Advanced Optical Manufacturing.

[159] Cui J., Zhao Q.C. (2024). Thermal stabilization of quality factor for dual-axis MEMS gyroscope based on joule effect In Situ dynamic tuning. IEEE Trans. Ind. Electron..

[160] Gill W.A., Howard I., Mazhar I., Mckee K. (2022). A review of MEMS vibrating gyroscopes and their reliability issues in harsh environments. Sensors.

[161] Goto K., Harada S., Hata Y., Ito K., Wado H., Cho J.Y., Najafi K. (2020). High Q-factor mode-matched silicon gyroscope with a ladder structure. Proceedings of the 2020 IEEE International Symposium on Inertial Sensors and Systems (INERTIAL).

[162] Bu F., Xu D.C., Zhao H.M., Fan B., Cheng M.M. (2018). MEMS gyroscope automatic real-time mode-matching method based on phase-shifted 45° additional force demodulation. Sensors.

[163] Marx M., Cuignet X., Nessler S., De Dorigo D., Manoli Y. (2019). An automatic MEMS gyroscope mode matching circuit based on noise observation. IEEE Trans. Circuits Syst. II Exp. Briefs.

[164] Zhang H., Zhang C., Chen J., Li A. (2022). A review of symmetric silicon MEMS gyroscope mode-matching technologies. Micromachines.

[165] Koenig S., Rombach S., Gutmann W., Jaeckle A., Weber C., Ruf M., Grolle D., Rende J. (2019). Towards a navigation grade Si-MEMS gyroscope. Proceedings of the 2019 DGON Inertial Sensors and Systems (ISS).

[166] Xu Z.Y., Xi B.Q., Yi G.X., Wang D.W. (2020). A novel model for fully closed-loop system of hemispherical resonator gyroscope under force-to-rebalance mode. IEEE Trans. Instrum. Meas..

[167] Wu Y.X., Pei L. (2017). Gyroscope calibration via magnetometer. IEEE Sens. J..

[168] Yang H.T., Zhou B., Wang L.X., Xing H.F., Zhang R. (2018). A novel tri-axial MEMS gyroscope calibration method over a full temperature range. Sensors.

[169] Zhou J., Jiang T., Jiao J.W., Wu M. (2014). Design and fabrication of a micromachined gyroscope with high shock resistance. Microsyst. Technol..

[170] Cameron C.P., Imamura T., Devmalya C., Vukasin G., Alter A., Kenny T. (2020). Design comparison and survivability of epitaxially encapsulated MEMS disc resonating gyroscopes at high shock (>27,000g). Proceedings of the 2020 IEEE International Symposium on Inertial Sensors and Systems (INERTIAL).

[171] Miao T.Q., Li Q.S., Hu X.P., Wu X.Z., Wu W.Q., Xiao D.B. (2022). Virtual rotating MEMS gyrocompassing with honeycomb disk resonator gyroscope. IEEE Electron. Device Lett..

[172] Guan Y.W. (2017). Research on the Dynamic Coupling Characteristics and Vibration Sensitivity of MEMS Tuning Fork Gyroscopes. Ph.D. Thesis.

[173] Liu M.X., Fan Q., Zhao J., Su Y. (2021). A phase compensation method for MEMS quadruple mass gyroscope in zero bias drift. IEEE Sens. J..

[174] Wang P., Li Q.S., Zhang Y.M., Wu Y.L., Wu X.Z., Xiao D.B. (2023). Bias thermal stability improvement of mode-matching MEMS gyroscope using mode deflection. J. Microelectromech. Syst..

[175] Ren J.B., Zhou T., Zhou Y., Li Y.X., Su Y. (2023). A real-time automatic mode-matching method based on phase-shifted virtual Coriolis force for MEMS disk resonator gyroscope. IEEE Sens. J..

[176] Wang P., Li Q.S., Xu Y., Zhang Y.M., Xi X., Wu Y.L., Wu X.Z., Xiao D.B. (2023). Calibration of coupling errors for scale factor nonlinearity improvement in navigation-grade honeycomb disk resonator gyroscope. IEEE Trans. Ind. Electron..

[177] Sun J.K., Liu K., Yu S., Zhang Y.M., Xi X., Lu K., Shi Y., Wu X.Z., Xiao D.B. (2022). Identification and correction of phase error for whole-angle micro-shell resonator gyroscope. IEEE Sens. J..

[178] Cao H.L., Wei W.Q., Liu L., Ma T.C., Zhang Z.K., Zhang W.J., Shen C., Duan X.M. (2021). A temperature compensation approach for dual-mass MEMS gyroscope based on PE-LCD and ANFIS. IEEE Access.

[179] Ren C.H., Guo D.N., Zhang L., Wang T.H. (2022). Research on nonlinear compensation of the MEMS gyroscope under tiny angular velocity. Sensors.

[180] Din H., Iqbal F., Lee B. (2021). Design approach for reducing cross-axis sensitivity in a single-drive multi-axis MEMS gyroscope. Micromachines.

